# On the material dependency of peri-implant morphology and stability in healing bone

**DOI:** 10.1016/j.bioactmat.2023.05.006

**Published:** 2023-05-19

**Authors:** Stefan Bruns, Diana Krüger, Silvia Galli, D.C. Florian Wieland, Jörg U. Hammel, Felix Beckmann, Ann Wennerberg, Regine Willumeit-Römer, Berit Zeller-Plumhoff, Julian Moosmann

**Affiliations:** a Institute of Metallic Biomaterials, Helmholtz-Zentrum Hereon, Max-Planck-Str. 1, 21502, Geesthacht, Germany; b University of Malmö, Faculty of Odontology, Department of Prosthodontics, Carl Gustafs Väg 34, Klerken, 20506, Malmö, Sweden; c Institute of Materials Physics, Helmholtz-Zentrum Hereon, Max-Planck-Str. 1, 21502, Geesthacht, Germany; d University of Gothenburg, Institute of Odontology, Department of Prosthodontics, Medicinaregatan 12 f, 41390, Göteborg, Sweden

**Keywords:** Biodegradable implant materials, Bone mechanical testing, Implant stability, Synchrotron micro-computed tomography imaging, Digital volume correlation

## Abstract

The microstructural architecture of remodeled bone in the peri-implant region of screw implants plays a vital role in the distribution of strain energy and implant stability. We present a study in which screw implants made from titanium, polyetheretherketone and biodegradable magnesium-gadolinium alloys were implanted into rat tibia and subjected to a push-out test four, eight and twelve weeks after implantation. Screws were 4 mm in length and with an M2 thread. The loading experiment was accompanied by simultaneous three-dimensional imaging using synchrotron-radiation microcomputed tomography at 5 μm resolution. Bone deformation and strains were tracked by applying optical flow-based digital volume correlation to the recorded image sequences. Implant stabilities measured for screws of biodegradable alloys were comparable to pins whereas non-degradable biomaterials experienced additional mechanical stabilization. Peri-implant bone morphology and strain transfer from the loaded implant site depended heavily on the biomaterial utilized. Titanium implants stimulated rapid callus formation displaying a consistent monomodal strain profile whereas the bone volume fraction in the vicinity of magnesium-gadolinium alloys exhibited a minimum close to the interface of the implant and less ordered strain transfer. Correlations in our data suggest that implant stability benefits from disparate bone morphological properties depending on the biomaterial utilized. This leaves the choice of biomaterial as situational depending on local tissue properties.

## Highlights

• Push-out test of screw implants imaged dynamically in 3D using micro computed tomography.
• Quantitative comparison of bone implant stability for titanium, PEEK and biodegradable magnesium-gadolinium alloys.
• Implant stability depends on implant materials and bone morphology-dependent load transfer.

## Graphical abstract

**Figure:**
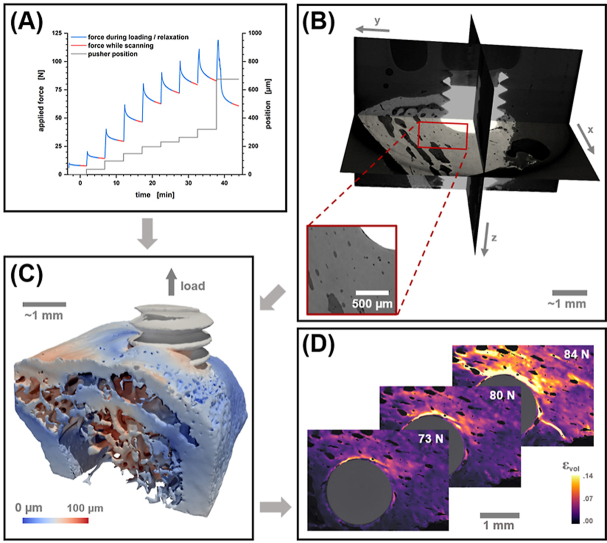

## 1 Introduction

Humans, both male and female, reach their peak bone mass between the age of 30 and 40. Thus, in the second half of our lives bone loss predominates over bone formation [1]. Estrogen deficiency in women during menopause accelerates the rate of bone loss even further and in an aging society bone fractures are a frequent occurrence. Any surgery is stressful to the patient and associated with risks that implantology should seek to minimize. This is especially true for elderly patients who are exposed to poorer healing outcomes and a higher morbidity after surgery [2]. For several decades, titanium has been referred to as the gold standard for implant materials [3]. Yet, one size does not fit all and the implant of the future should be more customizable, both in shape and the material it is made of [4]. The ideal material is biocompatible, integrates perfectly into bone and is readily replaced by newly formed bone tissue over time. Moreover, an elastic modulus close to the one of bone is desirable because it minimizes implant induced alterations in load distribution and tissue remodeling. Overly rigid biomaterials are susceptible to focusing stresses to the already compromised fracture location [5].

Most prominently titanium benefits from its capability to increase implant stability *via* osseointegration. Within a brief time, direct contact is made between implant surface and biological tissue as the former is colonizable by osteoblasts. In addition, titanium provides a high fracture toughness and longevity while being lightweight, which are favored properties to look for in a permanent implant [6]. However, using a material with an elastic modulus that is several times higher than that of natural bone comes at the cost of potential stress shielding. A local loss of bone density may be the consequence of such unfavorable stress stimulation on bone remodeling. Other factors to consider are the accumulation of long-term wear [3] and tissue calcification from foreign body reactions [7,8].

Implants made of polyetheretherketone (PEEK) seek to overcome the stress shielding effect of titanium-based implants by providing a Young's modulus that is close to the modulus of natural bone. Yet, PEEK does not integrate as well into existing tissue as titanium. This drawback may be partially overcome by the use of superficially porous PEEK [9]. The increased surface area that is offered to the surrounding tissue does not alter the bioactive properties of PEEK, i.e., it does not help to promote direct bone bonding. Yet, the bone–implant interface may be strengthened through additional mechanical interlocking.

Biodegradable implants, including the promising implants made of rare-earth alloyed magnesium, corrode when exposed to biofluids. This process increases the surface area available to the bone tissue over time and allows for potentially improved secondary implant stability *via* bone ingrowth [10]. Ultimately, a targeted release of ions that support hydroxyapatite formation may accelerate patient recovery. This may come in conjunction with a faster resolution of the initial inflammatory response [11]. Yet, for the time being, the immediate benefit of a resorbable implant lies in the possibility of reducing patient trauma – especially in pediatric applications – by eliminating the need for surgical explantation. Controlling the speed of degradation is one of the major challenges in the application of biodegradable implant devices and too rapid corrosion of Mg-implants is accompanied by uncontrollable hydrogen release. Hydrogen bubbles may result in adverse biological reactions and likely hinder bone ingrowth [6].

The extracellular matrix of bone itself is a complex composite comprised of predominantly hydroxyapatite and collagen. The material is hierarchical and heterogeneous and as such difficult to capture in a computational mechanical model where continuum assumptions need to be made on some scale. Hence, thorough experimental evaluation of the mechanical integrity and morphology of peri-implant bone via microimaging are invaluable tools in addressing bone–implant systems and calibrating future *in silico* modeling approaches [12]. Recently, the combination of micrometer scale imaging capabilities of synchrotron micro computed tomography (μ-CT) with *in situ* tensile testing devices for millimeter sized bone samples [[13], [14], [15], [16]] and bone–implant systems [[17], [18], [19], [20], [21], [22]] have become more feasible. Such an integrated setup extends the macroscopic load–displacement response acquired from a traditional *in vitro* pull-out or push-out experiment to a localizable mechanical response on the micrometer scale [20]. As pointed out by Le Cann et al. this is highly desirable because the complexity of the hierarchical bone structure entails vast uncertainties on the local mechanical response. Morphometric measures available do not provide a clear relationship between morphological parameters and implant stability [20]. By combining μ-CT imaging with a mechanical pull-out test the authors identified characteristic crack patterns around titanium screws four weeks after implantation into the tibial metaphysis of Sprague Dawley rats. In a follow-up study they described these cracks as a persistent crack envelop 300–500 μm from the implant–bone interface using digital volume correlation (DVC) software at 100 μm voxel resolution. They concluded that resolving the finer processes taking place would require higher resolution [19].

Push-out tests with pin-shaped implants made of magnesium alloys were performed by Tschegg et al. in 2011 on rat femur [10]. They reported considerable higher maximum push-out forces for the biodegradable implants than for their references made of titanium. A preceding study of Krüger et al. on magnesium degradation rates revealed that the average peri-implant bone morphological parameters for implants made of magnesium-gadolinium alloys would differ from the ones observed with non-degradable implants but their dataset lacked information on the underlying implant stability [23]. Therefore, it remained unclear if the observed morphological descriptors relate to implant stability and how the excellent stabilities reported for pin implants extend to screws. Here, we address these questions by taking a unified look at the local micrometer scale morphological and mechanical evolution of bone–screw implant systems over an array of 42 samples. The samples are composed of three conceptually different implant materials (titanium, PEEK and biodegradable magnesium-gadolinium alloys (Mg-xGd with x = 5 and 10 wt%)) with varying healing periods of four, eight and twelve weeks. We base our analysis on recordings made with absorption contrast synchrotron-radiation μ-CT imaging at 5.4 μm resolution combined with *in situ* mechanical testing *via* sequential push-out tests and calculated strain maps from these load sequences using a high-performance variational DVC solver operating on the voxel level. The experiments highlight a strong dependency of bone tissue related morphological measures in the 1 mm peri-implant region on the deployed biomaterial. This affects the strain transfer to the bone and potential correlations with morphometric measurements.

## 2 Methods

### 2.1 Implantation and sample preparation

The implantation protocol was previously described in Ref. [24]. In summary, male adult Sprague Dawley rats with an average weight of 350 g were selected for the study. Following anesthesia, an undersized osteotomy with final diameter of 1.6 mm was drilled into the tibia metaphysis. The rats were then implanted into both legs, either with two Mg-based (one Mg–10Gd and one Mg–5Gd) screws or two non-Mg screws (PEEK and titanium). PEEK and titanium screws were purchased from Promimic AB (Mölndal, Sweden) whereas Mg-based screws were custom turned [25]. All implants were used with an untreated surface and designed with an M2 thread that was evaluated with an effective height of 0.5 mm and an effective pitch of 0.4 mm from our data (see Figure SI1). All screws had a cut-off point, an effective major diameter of 1.9 mm and a length of 4.0 mm. The implants were manually screwed into the bone attempting a monocortical fit. Following four, eight, and twelve weeks of healing, the euthanasia of the rats was performed with a lethal dose of anesthetic. Subsequently, the legs were dissected and a 6 mm cylindrical region of bone surrounding the implant was extracted. These explants were frozen until the time of the experiment. Explants were kept hydrated prior and during the experiments.

All explants were mounted in a custom 3D-printed sample holder such that the actuator pin pushed paraxially to the principal axis of the screw implant. For this purpose, fast scans of each explant were acquired with a laboratory X-ray CT-scanner (Nanotom S, General Electric, Boston, United States) and transferred into 3D printable surface models. The outer surface of the explants was fixated by inserting a dimensionally stable resin-based composite filling (easyform LC, DETAX GmbH & Co. KG, Ettlingen, Germany) into the sample holder. The procedure was already described elsewhere in detail [17,18].

In total, 50 explants were used for acquiring *in situ* loading sequences resulting in 45 datasets of sufficient quality to be used for morphological analysis. Among those samples 17 came with a titanium implant of which 6 were harvested after four weeks, 5 after eight weeks and 6 after twelve weeks. We each had 11 datasets available with PEEK screws and Mg-10Gd screws both with 3 datasets after four weeks, 3 after eight weeks and 5 after twelve weeks. Mg-5Gd exhibited the highest failure rate with only 6 successful evaluations of which 3 were acquired after four weeks, 2 after eight weeks and 1 after twelve weeks. 36 of these samples could be evaluated for their nominal stiffness and 42 samples were used for identifying correlations as specified in the associated tables where relevant.

### 2.2 Mechanical testing and *in situ* CT-scanning

A graphical outline of the combined *in situ* push-out and synchrotron-radiation μ-CT imaging is provided with Figure SI2. Samples were mounted in a custom load frame that was designed for the simultaneous acquisition of tomographic data [18]. It incorporates an actuator suitable for applying forces of up to 1 kN to the samples. An initial tomographic reference scan of each sample was recorded after preloading the samples with up to 10 N of applied force and leaving them to equilibrate for 3 min. After each scan the load was increased stepwise following a protocol depending on healing period until catastrophic failure of the bone–implant system was observed and the samples were left to equilibrate again for 3 min (see Figure SI2A). Tomographic imaging was performed at beamline P05 operated by the Helmholtz-Zentrum Hereon at PETRA III at Deutsches Elektronen-Synchrotron (DESY, Hamburg, Germany) using conventional absorption contrast with a photon energy of 40 keV. 1200 projections were acquired from a 180° fly-scan with an exposure time of 34 ms per projection. Images were acquired using an indirect detector system with a CdWO4-scintillator and a 5120 x 3840 pixel CMOS camera located 4.7 cm from the sample. The microscope optics introduced a fivefold magnification resulting in an effective pixel size of 1.28 μm.

### 2.3 Dose considerations

The mechanical properties of bone are altered by exposure to radiation through the deterioration of collagen fibers which become increasingly brittle through non-enzymatic cross-linking [26]. Therefore, the dose the samples are expected to experience requires some serious consideration. According to Barth et al. the mechanical integrity of human bone, i.e., macroscopic strength, ductility and fracture toughness, does not experience any major changes for a dose of 35 kGy or less [26]. Peña Fernández et al. suggested an identical safe dose of 35 kGy for ovine trabecular bone [27]. In a previous study Moosmann et al. related these findings to rat tibia identifying a deterioration of mechanical properties above a radiation dose of 30 kGy [18]. After reducing the photon flux by a factor of 100, they estimated the dose experienced by the sample in our setup to be on the scale of 3.6 kGy for a scan with 1200 projections at 40 keV and 50 ms exposure. Thus, for being able to perform ten to twelve scans without major changes in mechanical properties we opted to reduce the exposure to 35 ms which equates to an expected effective dose of ∼2.5 kGy per tomogram. The obvious drawback is a decreased signal-to-noise ratio. Photon counts at the detector through samples incorporating a titanium-based implant were found to be especially low with only 1.6 times the average count identified in the darkfield images requiring thorough image processing (see Figure SI3) [15].

### 2.4 Image reconstruction and processing

Prior to tomographic reconstruction the flat- and darkfield corrected projection images were binned by a factor of four resulting in image volumes of 1280^3^ voxels at 5.1 μm voxel resolution close to the 5.4 μm spatial resolution of the imaging setup that was determined from the modulation transfer function. Reconstruction was performed with a MATLAB (R2020b, The MathWorks, Inc., Massachusetts, United States) script available at beamline P05. It implements the backprojection algorithm from the ASTRA toolbox with a Ram-Lak filter [[28], [29], [30], [31]]. Considering that the detector counts were comparably low, even errors in the approximation of the darkfield would result in pronounced ring artefacts. We found that these ring artefacts were best handled in post-processing using the Fourier transform based remove_ring filter that comes with the TomoPy library addressing rings of up to ten voxels width [32]. Additional streak artefacts and an overall higher noise level demanded a thorough denoising routine to not have artefacts bias the DVC calculations performed. This was especially crucial for the samples with a titanium-based implant. Best results were achieved by first targeting the random noise component with the Noise2Inverse filter followed by iterative non-local means filtering addressing textured noise components (see Figure SI4) [[33], [34], [35]]. A network for the former filter was trained using additional calibration scans recorded with 2400 projections at the end of a loading experiment. Segmentation into void phase, bone phase and implant material was performed on the initial scan of each loading sequence, referred to as reference scan later on. We used a custom seeded watershed algorithm to identify the interface with the maximal gradient magnitude between manually refined seeds for segmentation. This minimizes the influence of local variations in greyscale intensity on the final segmentation result. A graphical overview of the entire applied post-processing routine is provided with Figure SI5.

### 2.5 Macroscopic parameters, microscopic morphological analysis and statistical evaluation

Even with the 3D printed sample holders non-deformative micromotion cannot be fully eliminated, i.e., the apparent stiffness during the early stages of loading is reduced when the sample is pushed into the holder. Thus, we defined the nominal (elastic) stiffness by the maximal gradient in the force-displacement profile of the samples between two sequential scans. The gradients were determined by reading out the displacement of the actuator pin and the maximal applied force from the load cell at scan time.

For determining consistent bulk morphological parameters, a region of interest (ROI) was defined for each reference scan as the convex hull that would cover the biggest six-connected cluster of voxels labelled as bone. Additionally, the region was required to have a maximal distance of 630 voxels from the center of rotation of the reconstruction and to end above any reconstructed slice containing the highly absorbing actuator pin. The former condition safely excludes voxels reconstructed with incomplete angular information. Voxels outside this ROI were disregarded in the calculation of morphological parameters except for the exterior screw volume (V_ext_) for which all voxels marked as implant material above the ROI were summed up. Thus, the provided bone interface contact (BIC) does depend less on implantation depth and more on cortical thickness and number of trabeculae. It was calculated directly from the segmented three-dimensional image volumes as the ratio of voxel faces that describe a bone–screw interface to the sum of all interface voxel faces found in the material phase considering a six-connected neighborhood. Likewise, the absolute bone interface area in contact with the implant (A_BIC_) was taken as the sum of voxel faces describing a bone–screw interface. For the calculation of the bone area to bone volume fraction (BA/BV) the more accurate marching cubes surface area of the bone was taken into consideration [36], excluding interfaces at the boundaries of the ROI and to the implant. The measure was then derived by dividing the surface area of the bone by the volume of all voxels labelled as bone within the ROI. Mean positive normal curvature of the bone phase (κ_norm,pos_) was evaluated by estimating the local curvature of the bone at the bone–void interface within a spherical region of ten voxels radius. κ_norm,pos_ was then defined to result from the arithmetic mean of all curvatures greater zero within the ROI. The measure does not discriminate between trabecular and cortical tissue. Yet, on the scale of imaging the curvature of cortical bone is minor whereas trabeculae are highly curved. Mean bone volume to total volume fraction (BV/TV) in the ROI was derived from the ratio of voxels labelled as bone to the count of all voxels in the ROI, excluding implant material phase. For the calculation of two-dimensional local morphological properties measures were binned into radial profiles with respect to the implant's major axis. Constant variance in the measurement was assured by dividing the Euclidean distance maps into bins of approximately the same volume rather than bins of the same width. Distance in the radial profiles is either given with respect to the implant interface or a convex hull around the implant that we termed implant hull. 200 bins were used in the former, 250 bins in the latter case yielding bins close to the voxel size and covering a distance of up to 1 mm from the implant.

Correlations with bulk morphological parameters were calculated as adjusted Pearson Correlation Coefficients according to Ref. [37]. The significance of the adjusted correlations was evaluated at the 5% significance level. Locally resolved correlations of the BV/TV were evaluated equivalently after interpolating the BV/TV profiles onto a uniformly spaced distance axis.

### 2.6 Digital volume correlation and strain analysis

After an initial coarse alignment by rigid body registration onto the sample holder, local strains were determined using a custom digital volume correlation (DVC) software implemented with CUDA-C++. The software resolves the dense 3D optical flow of reconstructions from the samples under load (Frame 1) with respect to their corresponding reference scan (Frame 0). The implemented algorithm provides a further development to previous works on synchrotron-radiation μ-CT data [18,38,39] in which the identification of a dense 3D displacement field is formulated as a global variational problem. By extending the inner/outer iteration scheme introduced by Brox et al. [40] to 3D and incorporating a successive over-relaxation optimization scheme as described by Liu in 2D [41], the displacement field between two synchrotron-radiation scale volume images can now be evaluated on a GPU within minutes.

The energy functional to be minimized is defined with a data term enforcing brightness constancy between the frames and a smoothness term for regularization. We make sure that the critical brightness constancy assumption is fulfilled by normalizing 99.9% of the dynamic range in the two frames to a zero to one intensity range and applying a linear transformation that maps the local extrema in the histogram of Frame 1 onto the corresponding extrema in the histogram of Frame 0. Taking the square root of the input data further increases robustness because additional weight in the minimization of the data term is put on the lower intensity contrast between deformable bone and void phase. This reduces the overall weight of mismatches with larger intensity contrast, especially in the rigid and bright titanium phase.

An anisotropic flow-driven regularizer was chosen as a smoothness term [42] allowing for abrupt changes in the displacement fields. Such changes are expected to occur with detachments from the implant and cracking of the bone. The smoothness term was given a weight of 5% and implemented as a robust concave penalty term of the form $\Psi \left(s^{2}\right)=\sqrt{\left(s^{2}+\varepsilon^{2}\right)}$, where *ε* is a small constant and *s* stands for the smoothness term [40]. A spatial convolution was applied to the data term. This implements the combined local-global approach and thereby provides a solution that is more robust in poorly textured image regions with little local information content [43]. We chose the optimal 7-tap interpolation kernel of Farid and Simoncelli for 3D convolution [44]. Likewise, all other spatial derivatives were calculated with an optimal 5-tap stencil [44] since higher order derivatives were already shown to improve the quality of local DVC calculations (although with diminishing return) [45].

For the calculation of the deflection of the bone with respect to the implant and for visualization of deformations, all DVC determined displacement vector fields were converted into deformation vector fields by minimizing rigid body motion on the implant surface. After that transformation bone deflection was obtained as the mean volume of the deformation vector component parallel to major axis of the implanted screw. Volumetric strains ε_vol_ and the Green-Lagrange strain tensor were calculated from the deformation gradient *F* after introducing a spatial convolution to the vector components with a three sigma Gaussian kernel as ε_vol_ = det(*F*)-1 using a fourth-order finite difference scheme. 3D maximal principal strains were then calculated from the invariants of the Green-Lagrange strain tensor according to Ref. [46].

## 3 Results

**Fig. 1:**
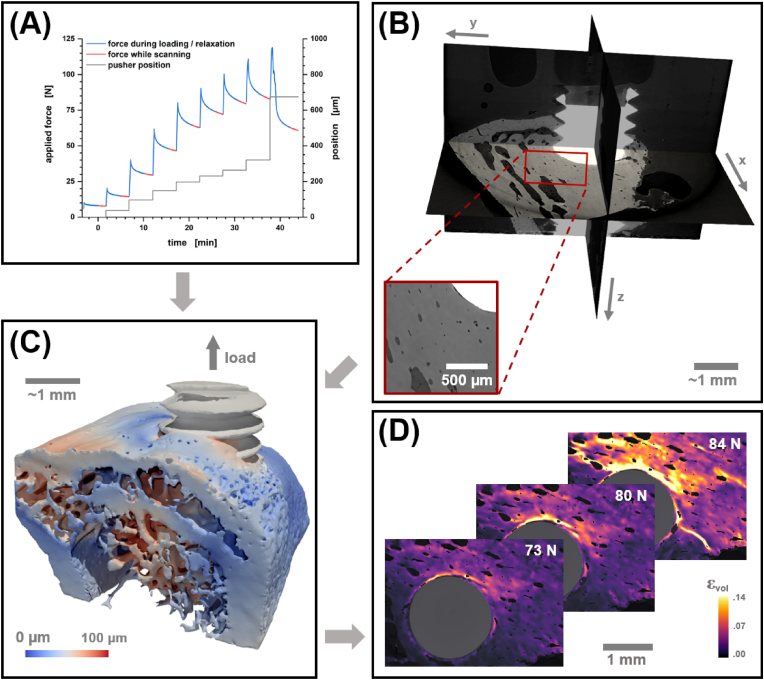
Illustration of the information obtainable from a push-out experiment combined with *in situ* μ-CT imaging and DVC analysis using an explant with a titanium screw as an example. (**A**) The applied force (blue/red line) and position of the pusher (grey line) are recorded from the load cell. (**B**) Micrometer scale 3D reconstructions, as shown by an orthoslice view, enable the morphological characterization of the peri-implant bone macrostructure. A detail view of the bone is provided showing vessels and osteocyte lacunae in the tissue. (**C**) Deformation fields are obtained from sequential scans at increasing load. This is depicted by a rendering of the peri-implant bone deformation magnitude. (**D**) Local strains and cracks in the bone are then accessible by image analysis as shown for an emerging longitudinal crack by plotting a sequence of 2D slices displaying volumetric strain at increasing load.

The information we acquire from *in situ* imaging of a bone push-out experiment is threefold: (i) There is the macroscopic mechanical response in the form of time-dependent force and displacement data that is tracked from the load frame while (ii) the local micrometer scale morphology of the peri-implant bone can be characterized from 3D reconstructions of the projection data (see Fig. 1A and B). (iii) Lastly, having a sequence of reconstructed states under increasing load allows quantifying local strains by determination of a deformative vector field that connects the observed states which is the objective of DVC (see Fig. 1C and D). The combination of spatially resolved local information and macroscopic mechanical response results in a setup that is fitting for determining structure–property relationships.

μ-CT resolves bone architecture solely at the macrostructural level of cortical and trabecular bone. This is the scale on which continuum mechanics take place and we might expect that the structural morphology on this scale is a determinant for the structural integrity of the bone and the implant stability. Unfortunately, this is a simplification that ignores the hierarchical built-up and composite nature of bone. E.g., the local proportion and arrangement of hydroxyapatite crystallites and collagen fibers in space as well as their molecular level build-up have a vast impact on the stiffness and ductility of the bone, resulting in a material of macroscopically heterogeneous mechanical properties [47]. An additional time-dependent factor is introduced from bone remodeling and maturation. It is entirely possible, that the lower-level information provided by the material composition might well be encoded in the higher-level structural features that we observe with μ-CT. However, we need to be aware that these unresolved features impose a limitation that reduces any attainable morphology–property relationships. Thus far, studies that consider implant stability in the context of macrostructural properties have reported mostly moderate to strong correlations [[48], [49], [50]].

Given the limitations described above, macroscale patterns in peri-implant bone tissue are realized and observable by μ-CT. In the following sections we attempt to put these patterns into an implant material dependent context.

### 3.1 Bone–screw implant stability and bulk morphological parameters

**Fig. 2:**
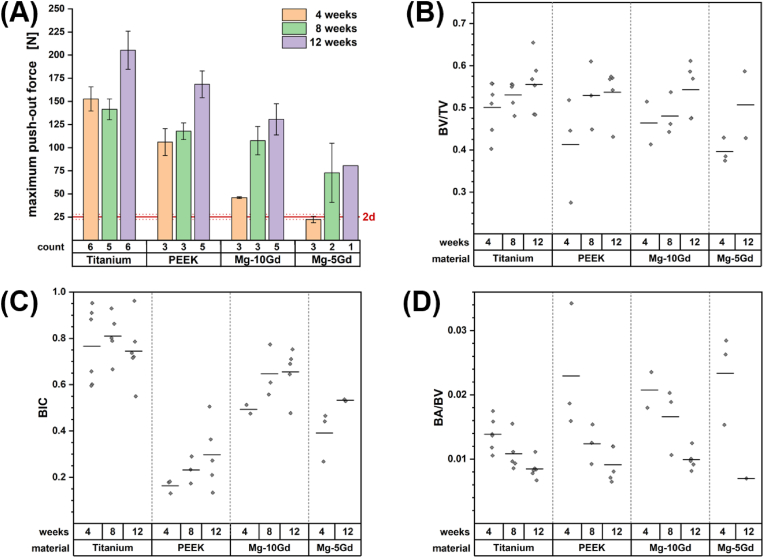
Illustration of the (**A**) expected value for the maximal applicable push-out force to 1.9 mm outer diameter screw implants and (**B–D**) statistical plots for the development of bone volume to total volume (BV/TV), bone interface contact (BIC) and bone area to bone volume ratio (BA/BV) depending on the implant material and the healing period. Error bars in panel **A** denote the standard error of the mean. The red line provides a lower boundary for primary implant stability. It is the average applicable force of two Mg-5Gd implants explanted after just two days. All statistical values in panel **B–D** were determined in a region of interest defined by the convex hull around the biggest connected cluster of segmented bone. Black lines mark the sample mean. Mg-5Gd samples after a healing period of eight weeks were excluded from analysis for lacking quality μ-CT reconstructions.

A measure of total implant stability is provided *via* the maximum push-out force (see Fig. 2A). A baseline for the primary implant stability was established at 25 N by considering two explants (incorporating an Mg-10Gd and an Mg-5Gd implant) that were acquired after just two days of healing. Naturally, the highest average push-out forces for any implant material were observed with a healing period of twelve weeks and explants incorporating an implant made of PEEK, Mg-10Gd or Mg-5Gd showed a progressive increase in implant stability after four, eight and twelve weeks of healing.

The highest implant stability was observed for titanium screws averaging a maximum push-out force of 205 N after twelve weeks and already 153 N after just four weeks. The conceptually similar study by Le Cann et al. that performed pull-out tests on titanium screws of 2.6 mm diameter implanted into the tibial metaphysis of Sprague Dawley rats for four weeks resulted into pull-out forces ranging from 40 N to 100 N [20]. Yet, higher failure loads in push-out tests compared to pull-out experiments are not uncommon [51]. The fast osseointegration and high biocompatibility of titanium are well-known [52] and the average bone volume fraction provided by the bone volume to total volume (BV/TV) and the bone–implant contact (BIC) support this (see Fig. 2B–C). BV/TV and BIC range among the most frequently reported image based bulk bone morphological parameters. Here, bulk describes the arithmetic mean of the respective morphological measure within the evaluated peri-implant region of interest. For titanium the bulk BV/TV is already 0.50 after four weeks of healing and improves progressively to 0.56 after twelve weeks. Other implant materials are found to be at a lower level of bone volume fraction during the early stages of healing. This agrees well with earlier findings of Krüger et al. who evaluated the BV/TV in the immediate vicinity of screw implants [23]. When their concept of a 100 μm–300 μm volume of interest (VOI) around the implant is adopted, our data can be considered to be derived from the same population in the majority of cases (see Table SI1). In both datasets, all bone–implant systems demonstrated a decreasing BV/TV with increasing VOI indicating callus formation.

BIC reflects the level of cellular adhesion on the implant surface. Thus, the rapid integration of titanium is expressed by high readings of the BIC that do not increase with longer healing periods (see Fig. 2C). With an attenuation coefficient that results in a rather low intensity contrast with respect to the water phase, segmentation uncertainties are surely increased for explants with a PEEK screw. Nevertheless, the very limited contact that we observe with these implants with a BIC of initially only 0.16 is in line with our expectations [23]. In fact, the BIC for PEEK is more easily overestimated with lower resolving CT scanners because image resolutions below 100 μm are required to capture the lack of cellular adhesion to the surface. It remains unclear whether a limited inflammatory response, the smooth surface of the polymer or its hydrophobicity cause the reduction in apparent cellular coverage [[53], [54], [55]].

In biodegradable implants the corroded regions attenuate less than uncorroded ones. This may result in reconstructed voxel intensities close to the ones found in bone tissue, which complicates the precise identification of the bone–implant interface with absorption contrast alone. Even more, as bone tissue grows into the degrading biomaterial over time and metallic species diffuse into the bone the greyscale gradient between the two phases expands and becomes increasingly fuzzy for longer healing periods. This increases the uncertainty in the BIC estimate. Comparisons between populations are additionally impeded by the fact that the domain around the implant is heterogenous. It is comprised of cortical bone, medullary cavity and biomaterial protruding from the bone. To minimize the dependency of the BIC on the implantation depth of the screw we decided to exclude the protruding part of the screw head from our BIC calculations. Variability in the measure was reduced by only considering the bone containing volume which necessarily results in higher readings on average than the values provided by Krüger et al. [23] but with the same trends (see Table SI1).

With Mg-5Gd implants close to half of our push-out experiments failed prematurely during preloading of the sample. With less than three evaluable samples for eight- and twelve-week healing periods we will not consider it in detail throughout our discussion. *In vitro* tests suggest that Mg-5Gd degrades several times faster than Mg-10Gd [56] which may have caused the frequent collapse of the implant that we observed upon preloading the samples. The difference in corrosion rates was not confirmed *ex vivo* presumably due to high standard deviations [23], but for the time being we regard the corrosion as too fast to pursue Mg-5Gd further as a material for screw implants. Ideally, the corrosion speed of the implant is matched to the growth of the bone. With the presumed slower corrosion of Mg-10Gd than for Mg-5Gd longer push-out sequences were obtainable. The observed average maximum push-out force of 131 N agrees very well with the push-out forces reported by Tschegg et al. after three months of healing for pins made from Elektron magnesium alloys [10]. Yet, in their experiments push-out forces for pins made of titanium alloys topped at approximately 100 N. This raises the question how the topography of a screw contributes to the stability of non-degradable implants. It is noteworthy, that a biodegradable magnesium alloy may be penetrated by bone tissue once it corrodes. This bone ingrowth appears to fixate a pin and a screw to a similar extent given sufficient time.

The visible bone in the ROI of our scans is dominated by the cortical bone that serves as an anchor for the implant. Yet, cortical bone near the implantation site frequently displays additional cavities and trabecular bone is present to a varying degree (see Fig. 1C). At 5 μm resolution even individual vessels and osteocyte lacunae are visible (see Fig. 1B). Therefore, we report the ratio of the bone area to the bone volume (BA/BV) as an approximate measure of the inverse cortical thickness that respects the mixed composition of the analyzed tissue, i.e., it contains both cortical and trabecular bone. The build-up and/or thickening of peri-implant cortical bone over time is observed to be monotonously increasing for all the implant materials studied. After twelve weeks of healing the mean of the BA/BV reduces to a value that is similar for the tested biomaterials (see Fig. 2D).

### 3.2 Material dependent peri-implant morphology

**Fig. 3:**
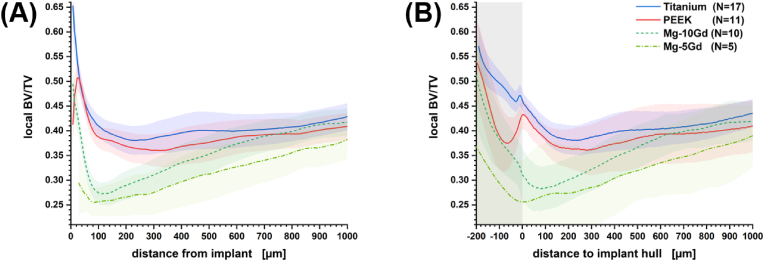
Radially resolved mean bone volume fraction for different implant materials (**A**) depending on the Euclidean distance from the implant surface and (**B**) depending on the Euclidean distance from the convex hull covering the implant. In the latter case negative distances, accentuated with a grey shade, are associated with the volume located between the screw threads. Blue, red and green shaded areas provide the t-score based 95% confidence interval.

The properties of the biomaterials described previously have such a strong influence on the remodeling of the bone tissue that the differences in the bone volume fraction realized in the vicinity of the implant are far more pronounced with respect to the implant material than they are with respect to the extent of the healing period (see Figure SI6). Thus, it appears admissible to average the radial bone volume fraction over the samples at different healing periods for providing a standard profile that is characteristic for the implant material under consideration. We provide profiles with respect to the Euclidean distance to the implant interface and with respect to the distance to the surface of a convex hull covering the implant (see Fig. 3). In the latter case negative distances denote the volume between the screw threads that we expect to experience different mechanical stimuli than the bone volume exterior to the threads.

Mechanical stimuli accelerate tissue formation near the implant resulting in a bulging high-density region of bone tissue at the bone–implant interface for titanium and magnesium alloys. With passing time these layers thicken, i.e., increased readings of the BV/TV are found at a greater distance from the bone–screw interface (see Figure SI6). With PEEK as an implant material the highest volume density of bone tissue is found approximately 25 μm away from the implant (see Fig. 3A & Figure SI6D). This is certainly a consequence of the very limited *in vivo* cellular adhesion. Hence, we expect micro-motions to be more pronounced than with biomaterials that enable tissue coverage resulting in micrometer-scale gaps between organic tissue and implant. A local maximum in BV/TV is found precisely at the ridge of the screw threads (see Fig. 3B). The amount of tissue near the interface increases and occupies a higher volume ratio with progressing healing period but maintains its spatial extension of approximately 100 μm from the implant site (see Figure SI6D).

Magnesium ions are considered to promote osseointegration and tissue growth [57]. Therefore, it seems counterintuitive that implants made of biodegradable magnesium gadolinium alloys reliably exhibit a minimum in BV/TV next to the implant. For Mg-10Gd the minimum is located ∼70 μm from the implant after four weeks, ∼100 μm after eight weeks and ∼130 μm after twelve weeks of healing (see Figure SI6E). It does not coincide with the ridge of the screw threads. Thus, in interpreting this minimum we may also have to consider that the mechanical stimulus on the tissue changes with time as the material corrodes. Additionally, corrosion may lead to changes in the chemical environment and to the formation of hydrogen pockets at the bone–implant interface limiting tissue growth [57]. Altogether, remodeling of bone can be observed to extend further away from the implant than with non-resorbable biomaterials. While the BV/TV with titanium and PEEK suggests bulk behavior approximately 200 μm away from the implant interface magnesium-gadolinium alloys result in a gently inclined BV/TV profile at least 700 μm into the bone.

### 3.3 Material dependent strain uptake

With the availability of scans under load our analysis may extend beyond a mere morphological description of the bone tissue. DVC enables us to monitor the apparent strain distribution in a deformed sample. By using a variational solver on the voxel scale, strains and smaller cracks can be localized with high precision given sufficient textures provided by interfaces and resolved blood vessels (see Figure SI7). Cracks result in a strain singularity, i.e., the approach is certainly not fully quantitative and scans after catastrophic failure of the bone or implant had to be excluded from analysis.

**Fig. 4:**
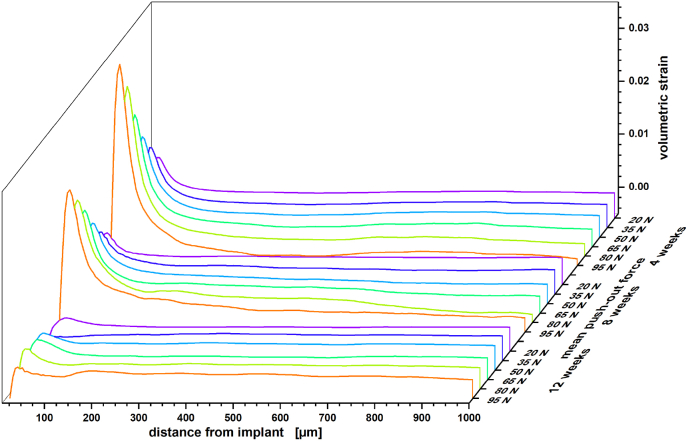
Mean volumetric strain for peri-implant bone of titanium implant systems with respect to healing period and applied force during scan time (color). Standard errors for the measure are provided with the Supporting Information (see Figure SI11).

Nevertheless, with titanium as an implant material we observed a highly consistent strain development in the vicinity of the implant. In Fig. 4 this is plotted in the form of volumetric strain depending on the Euclidean distance to the implant and with respect to the applied load and healing period. Le Cann et al. earlier reported a reproducible strain pattern around titanium screws that they would locate in close proximity of their implants using DVC elements of 100 μm [19]. With our scans we consistently find a maximum in volumetric strain 30–35 μm from the bone–implant interface. Before catastrophic failure, this strain maximum increases linearly by 0.039% per Newton load in samples acquired with a healing period of four weeks whereas distal regions are slightly but continuously compressed. As bone remodeling progresses and cortical bone thickens/stiffens less volumetric strain is transferred to the bone and the increase of the volumetric strain maximum reduces to 0.027% per Newton after eight weeks of healing and almost diminishes after twelve weeks.

**Fig. 5:**
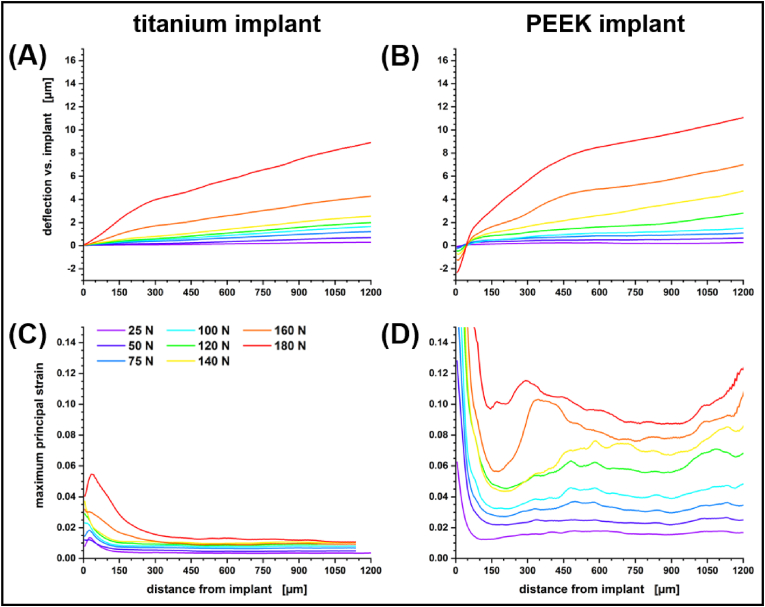
Comparison of the (**A**,**B**) deflection and (**C**,**D**) maximum principal strain for a titanium implant system (left panels) and a PEEK implant system (right panels). The titanium screw was implanted into the right tibia and the PEEK screw into the left tibia of the same rat for twelve weeks before explantation. Both implants failed at an applied force of 200 N, yet show vastly different patterns of strain uptake. These patterns appear to be representative for the implant types.

**Fig. 6:**
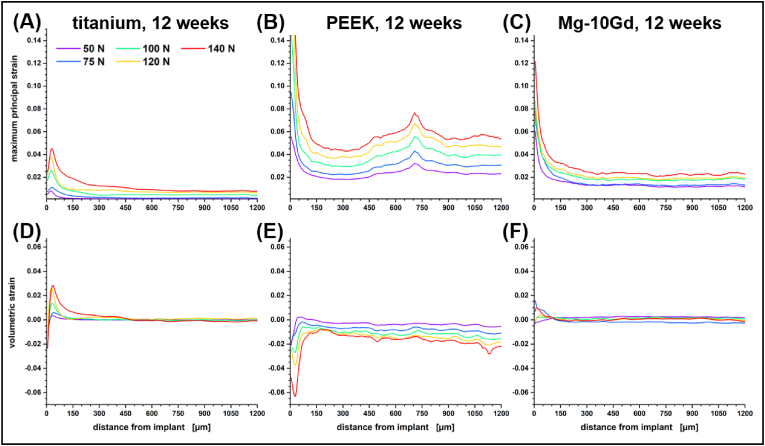
Comparison of **(A**–**C)** maximum principal strain and **(D–F**) volumetric strain in peri-implant bone with titanium implants (left panels), PEEK implants (central panels) and Mg-10Gd implants (right panels). All three implants were explanted after 12 weeks and failed at an applied push-out load of approximately 160 N.

With PEEK as an implant material the strain distribution in the bone tissue is decidedly different from the regular straining observed with titanium screws. The strain level itself is not as predictable, depending more on local architecture, but always markedly higher than with titanium. Fig. 5 compares the deflection of the bone and observed maximum principal strain for two twelve-week samples with PEEK and titanium implant. The samples were acquired from the left and right leg of the same rat. Both samples failed at ∼200 N applied load and are representative of the general behavior of the tibiae under load. With an elastic modulus of approximately 3.6 GPa the material properties of PEEK are close to that of rat femoral cortical bone [58]. Therefore, strain is more distributed and the whole system reacts more like a sponge. Strains are still highest at the screw–bone interface but are mostly compressive and remain high and extend evenly throughout the whole field of view without diminishing. With PEEK we always observed a negative deflection near the implant surface that increases with load, giving the appearance that the bone is being pushed-out further than the screw, but this is deceptive. For the calculation of the deflection we free the surface of the implant from any rigid body motion assuming an undeformed implant. This is a safe assumption for the metallic implants and the better part of deformative work always takes place in the bone pressed into the rigid sample holder. Yet, for a material as soft as PEEK reactive forces at loads above 100 N are large enough to induce a measurable sub-voxel scale compression of the implant (see Figure SI12). This is indicated by the apparent negative deflection of the bone with respect to the implant surface that is assumed to be stationary. The presence of negative volumetric strains near the implant surface supports this interpretation (see Fig. 6 & Figure SI10). Note that the local strain maxima at higher applied forces in Fig. 5 indicate the onset of failure through the formation of initial cracks in a 300–400 μm envelope around the sample (cf. [19]) resulting in an inconclusive volumetric strain profile for this particular sample (see Figure SI8).

The peri-implant strain profiles of bone tissue around Mg-10Gd implants are not entirely coherent (cf. Fig. 6C,F and Figure SI10C,F). More often than not the implant material yields before the bone tissue. This starts with a collapse of degraded regions. Thus, strain transfer to the bone is frequently rather small and does not necessarily increase or decrease monotonically locally (cf. Fig. 6C,F & Figure SI10C,F). The uncorroded material has a Young's modulus of 43.6 GPa [56] which reduces the amount of stress shielding compared to titanium with an elastic modulus of more than twice that value. Nevertheless, Mg-alloys are stiffer than mineralized bone and strain maxima 30–35 μm from the interface do appear in the peri-implant strain profiles of Mg-10Gd samples but with smaller amplitudes than observed for titanium (see Figure SI10C). In additional contrast to titanium, catastrophic failure does not always initiate from the jagged screw-tissue interface into the tissue. In implant–bone systems with corroded implant material, dislocation of the screw itself is the prevalent failure mode and metallic residuals can be made out on the previous interface location marking the ingrowth of bone tissue (see Figure SI9).

### 3.4 Correlations of implant stability with bone morphological parameters

It is to be expected that the measured stiffness of the bone–implant systems ranges among the best descriptors for implant stability (see Table 1). Without considering any material dependency we found that screw implants in rat tibia would exhibit a nominal stiffness of 3.5·10^2^ N/mm after four weeks of healing. Stiffness increases to an average of 4.2·10^2^ N/mm after eight weeks before reaching 4.8·10^2^ N/mm after twelve weeks (see Table SI2). Nominal bone stiffness with metal implants increased with healing period and twelve weeks after implantation, titanium implants averaged at 6.1·10^2^ N/mm and Mg-10Gd implants at 4.9·10^2^ N/mm. PEEK implants displayed decidedly less stiffening in the peri-implant bone tissue reaching 3.6·10^2^ N/mm after twelve weeks. The correlation with the observed maximum push-out force is strong to very strong in almost all groups considered (see Table 1). Unfortunately, a bone stiffness measure is comparably hard to acquire in clinical praxis and morphological descriptors that can be extracted from a single CT scan would be much preferred.

**Table 1:** Adjusted correlations for the mean stiffness (${\bar{k}}_{\text{nom}}$) with maximum push-out force (F_max_) and with bulk morphological descriptors of the unstrained bone–implant domain. Significant correlations are marked with a star.

Material	Time	N	${\bar{k}}_{nom}$ [10^2^ N/mm]	F_max_	BV/TV	BIC	A_BIC_	V_screw,ext_	BA/BV^a^	κ_norm,pos_
Titanium	any	17	5.3	0.68*	0.48	−0.21	0.30	−0.48	−0.09	0.29
PEEK	any	9	3.1	0.93*	0.75*	0.40	0.33	0.32	−0.66	−0.77*
Mg-10Gd	any	9	3.8	0.98*	0.21	0.65	0.57	−0.72*	−0.90*	−0.58
any	4 weeks	11	3.5	0.94*	0.29	0.68*	0.66*	0.34	−0.46	−0.36
any	8 weeks	11	4.2	0.96*	−0.20	0.30	0.56	−0.53	−0.43	−0.02
any	12 weeks	15	4.8	0.67*	−0.16	0.63*	0.57*	−0.18	−0.30	−0.03
Titanium	4 weeks	6	4.7	0.75	−0.44	0.06	0.21	−0.17	0.58	0.56
Titanium	8 weeks	5	5.2	1.00*	−0.82	−0.79	0.18	−0.95*	0.12	0.34
Titanium	12 weeks	6	6.1	0.51	−0.96*	0.18	0.20	−0.22	0.19	0.91*
PEEK	12 weeks	5	3.6	0.95*	0.85	0.19	0.03	0.38	−0.81	−0.97*
Mg-10Gd	12 weeks	4	4.9	1.00*	0.01	0.95	0.93	−0.89	−0.92	0.61
any	any	36	4.3	0.79*	0.13	0.48*	0.57*	−0.27	−0.50*	−0.27

* significant at p < 0.05.

a Interfaces to the screw and to narrow vessels were excluded from the evaluation of the surface area.

Seebeck et al. stated in 2005 that *“a sufficiently accurate prediction of the holding capacity of screws as a function of local bone morphology has not been obtained*” and to the best of our knowledge this is still true [59]. Therefore, we need to be careful when drawing conclusions from apparent correlations in our dataset as the number of samples in a study that involves live specimens necessarily remains small. Nonetheless, there are indications that are in line with our above analysis. Table 2 provides adjusted correlations [37] for the maximum push-out force with morphological parameters that were evaluated from the initial scan of the samples preloaded with 5 N. The well-known BV/TV and BIC measures that provide ratios are complimented by additional measures calculated within the ROI. These are the absolute bone interface area in contact with the implant (A_BIC_), the exterior screw volume (V_screw,ext_) defined by the volume of screw material protruding outward from the cortical bone, bone area to bone volume ratio (BA/BV) and mean positive normal curvature of the bone phase (κ_norm,pos_). Across all 42 samples in our study BV/TV can be considered as a significant, yet moderately strong, descriptor of implant stability (r = 0.50) alongside κ_norm,pos_ (r = −0.53) and BA/BV as a strong descriptor (r = −0.65) that are both anticorrelated. BIC, A_BIC_ and V_screw,ext_ appear to be insignificant. This changes once we consider the morphology with respect to the material of the implanted screw.

**Table 2:** Adjusted correlations for the maximum push-out force with bulk morphological descriptors of the initial bone–implant domain. Significant correlations are marked with a star.

Material	Time	N	BV/TV	BIC	A_BIC_	V_screw,ext_	BA/BV^b^	κ_norm,pos_
Titanium	any	17	0.06	−0.30	0.19	−0.53*	−0.12	0.09
PEEK	any	11	0.84*	0.48	0.43	0.23	−0.82*	−0.88*
Mg-10Gd	any	10	0.43	0.78*	0.74*	−0.72*	−0.77*	−0.54
any	4 weeks	14^a^	0.50	0.51	0.39	0.47	−0.64*	−0.66*
any	8 weeks	11	0.06	0.27	0.26	−0.45	−0.55	−0.25
any	12 weeks	17	0.17	0.20	0.05	0.20	−0.11	−0.01
Titanium	4 weeks	6	−0.37	0.01	0.47	−0.90*	0.63	0.37
Titanium	8 weeks	5	−0.86	−0.96*	0.24	−0.97*	0.17	0.42
Titanium	12 weeks	6	0.03	−0.25	−0.18	−0.07	0.81	0.70
PEEK	12 weeks	5	0.84	0.14	0.05	0.16	−0.35	−1.00*
Mg-10Gd	12 weeks	5	0.30	0.90*	0.77	−0.76	−0.39	−0.26
any	any	42	0.50*	0.32	0.29	−0.06	−0.65*	−0.53*

* significant at p < 0.05.

a N = 13 for the calculation of the V_screw, exterior_ correlation.

b Interfaces to the screw and to narrow vessels were excluded from the evaluation of the surface area.

PEEK has the lowest Young's modulus among the tested biomaterials and it is the only material tested with a surface that is adversarial to the colonization by osteogenic cells. This already had a major effect on bone remodeling and strain uptake. When we look at all healing periods collectively, BV/TV shows a very strong correlation with implant stability, while BA/BV and κ_norm,pos_ exhibit a strong anticorrelation. This provides an indication that PEEK implants are primarily stabilized by the presence of thick cortical bone tissue because κ_norm,pos_ is expected to increase in the presence of trabecular bone tissue and BA/BV will behave anti-proportional to cortical thickness.

With Mg-10Gd as an implant material, κ_norm,pos_ and BV/TV appear to be less influential and strong correlations or anticorrelations of the maximum push-out force are observed with the BIC, A_BIC_, V_screw,ext_ and the BA/BV ratio. V_screw,ext_ is essentially an anti-proportional measure to the implantation depth. Thus, all four measures relate the holding capacity to the implant surface area that is in contact with the bone tissue, i.e., holding capacity increases with the amount of available interface area and bone ingrowth is a likely contributor to the observed total implant stability.

**Fig. 7:**
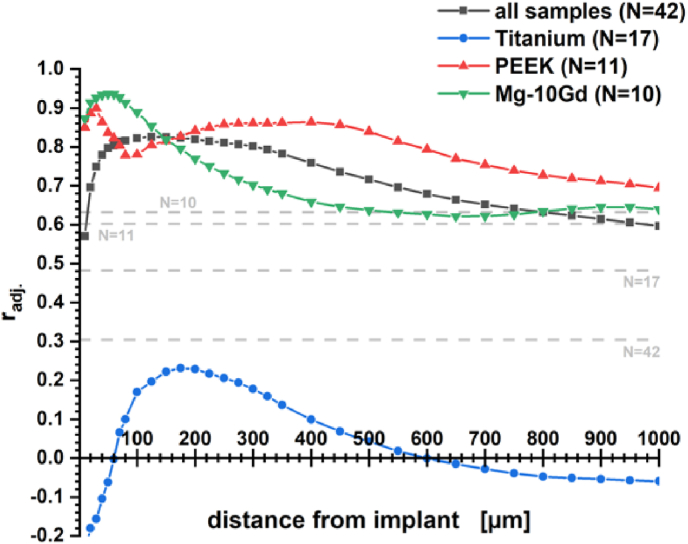
Correlation of local BV/TV with maximum push-out force. Grey lines correspond to the significance level at p < 0.05 for the given sample size.

For titanium implants our experiments do not result in a measure that would correlate implant stability with bone morphology. Especially, the correlation with BV/TV was only very weak, whereas Le Cann et al. earlier reported a strong correlation (r = 0.67) for more scattered pull-out forces with trabecular BV/TV for 2.6 mm screws four weeks after implantation into rat tibia [20]. It was highlighted by Wirth et al. in 2011 that BV/TV should be considered as a local rather than as a global parameter for evaluating implant stability [60]. In their modeling study, global bone density appeared as a moderately good descriptor for stiffness whereas local bone density was a very strong descriptor. The term local described a 7 mm scale for a virtual biopsy of human humeral heads. Fig. 7 picks up this idea by evaluating the correlation of experimentally observed maximum pushout force with the BV/TV at a given distance from the bone–screw interface. This provides an expression for what could be considered a local correlation in the scope of rat tibia. Especially, the correlation with the BV/TV measure for Mg-10Gd implants improved considerably showing a very strong correlation of 0.94 with the maximum push-out force at 50 μm distance. With PEEK the locality window widens to a local significance maximum at 400 μm putting more emphasis on overall bone material density than on contact area. For titanium screws a maximum in correlation was found 175 μm from the implant surface but remained insignificant.

Ultimately, V_screw,ext_ proved to be the only measure that was moderately anticorrelated with titanium implant stability across all three healing periods. The correlation is very strong after four and eight weeks but insignificant after twelve weeks of tissue remodeling, i.e., a deep implantation appears to be beneficial in the early stages of healing and potentially a decent proxy for insertion torque that we could not obtain. The latter was found to yield the strongest correlation with primary pull-out strength (r = 0.835) in a comprehensive correlative *post mortem* study by Marquezan et al. [48] with 1.4 mm diameter Ti–6Al–4V screws. Popular image based morphological descriptors such as the trabecular thickness (r = 0.578) and bone volume fraction (r = 0.553) were significant but only moderately strong correlated. Please note, that the very strong anticorrelation that we observe with the BIC after eight weeks is likely not beneficial to the implant stability itself but a misleading correlation. Interfaces to the cortex commonly exhibit more contact to the implant than interfaces to trabecular bone that are found in the medullary cavity. Uncovered biomaterial outside the bone is not considered in the BIC calculations. Hence, deeper implanted screws result in an increased relative contribution of contact area in the medullary cavity to the measure, i.e., lower BIC readings on average. In conclusion, we expect that the excellent stability observed for the bone-titanium systems is less a result of tissue adhesion but caused by the proliferation of tissue between the jagged implant threads resulting in additional mechanical stabilization through jamming.

## 4 Discussion

Our μ-CT study shows that biomaterials affect the remodeled peri-implant bone tissue in a variety of ways. We considered implants with disparate material properties and different healing periods and it has to be accentuated that the former affects the peri-implant morphology of newly formed bone to a greater extent than the latter. BV/TV and BA/BV measurements approached similar levels for PEEK, Mg-xGd and titanium implants after twelve weeks of healing when considered as an average ROI parameter. Yet, the radially resolved bone volume fraction proved to be characteristic for each implant material. Foreign body reactions and stress stimuli result in a proliferation of bone tissue near the interface to titanium implants. The same is true for PEEK implants, but as the tissue avoids direct contact with the implant, BV/TV between the screw threads is reduced. Magnesium-gadolinium alloys do show decreased callus formation. In fact, proliferation of tissue is only observed between the threads and a minimum in bone volume fraction appears reproducibly next to the implant.

When studying strain with X-ray imaging in biological tissue, care has to be taken with respect to dosage and image restoration. With that in mind, insights on the micrometer level strain distribution in bone–screw implant systems were obtained by DVC with micrometer scale precision. Cracks and fractures that develop under load appear as strain singularities in a DVC analysis and thus strain values should not be considered to be fully quantitative. Our analysis demonstrates experimentally how forces acting on an implant are transferred to the surrounding bone tissue. With an implant material like titanium that is substantially stiffer than bone tissue, local maxima in tensile strain are observed 35 μm from the implant interface. These strains diminish as the bone matures and stiffens. The acquired strain profiles from explants bearing a titanium implant proofed to be replicable for any fixed healing period and may therefore serve in calibrating mechanistic models in the future.

Matching the elastic modulus of bone tissue with PEEK results in a delocalized transfer of strains to the tissue and apparently less stiffening of the tissue over time. We expect local heterogeneities in the mechanical properties of surrounding bone tissue to have a larger impact on the resulting strain distribution profile which deterred us from providing an average standard profile. Yet, we do see that more distributed strains resulted in remodeled tissue that was more flexible (see Table 1) and that the density of tissue further away from the implant site contributed more to the stabilization of the implant than it did for metals (see Fig. 7). Negative strains at the bone–implant interface that were only observed with PEEK implants indicate that the organic material between the screw threads is compressed upon loading the implant (see Figure SI12).

Biodegradable implants transferred less strain to the tissue and the onset of fracture originated from the corroded regions of the implant rather than from the surrounding tissue. Stiffening of the bone was found to be higher than for PEEK but lower than for titanium. The readings in BA/BV after twelve weeks are coherent for all biomaterials tested. Yet, there is a lack of significant correlations with stiffness in our data. Thus, thickening of cortical bone at the implant site may increase the apparent stiffness of the tissue but disparities between biomaterials need to be explained by other parameters, e.g., the bone material density which cannot be reliably determined from the available absorption data.

Implant stabilities observed for Mg-10Gd screw implants conform very well with the stability values reported for Mg-alloy pin implants by Tschegg et al. [10]. However, Tschegg et al. also reported substantially lower push-out forces for titanium pins which we cannot confirm with our data for screw implants. This implies that for a titanium screw implant mechanical stabilization through interlocking of proliferating tissue with the threads is a bigger contributor to the implant stability than biological stabilization through cellular adhesion. Such interlocking certainly also benefitted the PEEK implants. Yet, depending on the healing period, titanium screws outperformed PEEK screws, that do not experience any additional biological stabilization, by 23 N–47 N. This is a comparable increase as observed between pins made from poly(lactic-co-glycolic) acid and titanium by Tschegg et al. [10] and may well be the level of stabilization that can be associated with osseointegration through adhesion. In contrast to titanium, bone ingrowth appears to be the driving force for the implant stability observed with Mg-alloy based biomaterials. Hence, it seems reasonable that morphological measures of available interface area are fairly strong correlated to the observed implant stability of Mg-10Gd screw implants. Mechanical stabilization is less important because implant failure originates from the screw and the callus does not extend beyond the screw threads as is indicative by a reduced BV/TV in the peri-implant region. The connection strength between the metal and the degradation layer of degrading implants may pose an upper limit to the implant stability upon direct loading of the implant. Yet, decent stabilities can be obtained through ingrowth at smooth interfaces reducing the need for drilling and cutting mechanical anchor points in inconvenient and sensitive tissue locations.

Ultimately, the material related correlations that we observed for implant stability may provide guidance in evaluating a fracture location for a suitable implant material. Our data suggest that titanium is best suited for a fracture that allows for deep implantation with a decent mechanical anchor. PEEK implant stability correlates with cortical thickness and therefore these materials seem to perform best in the presence of a healthy cortical bone. Degradable implants on the other hand may be advantageous when a shallow implantation is required, the topology of the fracture is either fringy, offering plenty of surface area, or extremely smooth, offering little or no mechanical anchor point. Bone–implant contact area is the strongest contributor to implant stability and thus design of a biodegradable should not target a sleek shape but focus on maximizing surface area. Because of this particular behavior, future studies should compare different biodegradable Mg-based implants amongst each other to optimize their overall mechanical performance ultimately.

## Ethics statement

The animal experiments were approved by the ethical committee at the Malmö/Lund regional board for animal research, Swedish Board of Agriculture (approval number DNR M 188-15). The authors declare no competing interests.

## Data availability statement

The datasets and source code generated and analyzed during the study are available from the corresponding authors upon reasonable request.

## CRediT authorship contribution statement

**Stefan Bruns:** performed the experiments, processed and, Formal analysis, all data and discussed them, wrote the manuscript. **Diana Krüger:** prepared the samples. **Silvia Galli:** prepared the samples. **D.C. Florian Wieland:** performed the experiments. **Jörg U. Hammel:** developed the experimental setup. **Felix Beckmann:** developed the experimental setup. **Ann Wennerberg:** prepared the samples. **Regine Willumeit-Römer:** conceived and, Supervision, the project. **Berit Zeller-Plumhoff:** performed the experiments, conceived and, Supervision, the project, processed and, Formal analysis, all data and discussed them, conceived and supervised the project. **Julian Moosmann:** developed the experimental setup, set up and optimized the experiment and provided reconstruction tools, performed the experiments, conceived and, Supervision, the project.

## Declaration of competing interest

The authors declare that they have no known competing financial interests or personal relationships that could have appeared to influence the work reported in this paper.
