# Supplementary material for: On the material dependency of peri-implant morphology and stability in healing bone
Source: Bioact Mater. 2023 May 19;28:155–66. doi: 10.1016/j.bioactmat.2023.05.006 (PMC10212791; doi:10.1016/j.bioactmat.2023.05.006)
Supplement: Multimedia component 1 [file mmc1.docx]

**Supplementary Information** **to**

**On the Material Dependency of peri-Implant Morphology and Stability in Healing Bone**

Stefan Bruns^1,*^, Diana Krüger^1^, Silvia Galli^2^, D.C. Florian Wieland^1^, Jörg U. Hammel^3^, Felix Beckmann^3^, Ann Wennerberg^4^, Regine Willumeit-Römer^1^, Berit Zeller-Plumhoff^1,*^ and Julian Moosmann^3^

*^1^ Institute of Metallic Biomaterials, Helmholtz-Zentrum Hereon, Max-Planck-Str. 1, 21502 Geesthacht, Germany*

*^2^ University of Malmö, Faculty of Odontology, Department of Prosthodontics, Carl Gustafs Väg 34, Klerken, 20506, Malmö, Sweden*

*^3^ Institute of Materials Physics, Helmholtz-Zentrum Hereon, Max-Planck-Str. 1, 21502 Geesthacht, Germany*

*^4^ University of Gothenburg, Institute of Odontology, Department of Prosthodontics, Medicinaregatan 12 f, 41390, Göteborg, Sweden*

*Correspondence and requests for materials should be addressed to Stefan Bruns (stefan.bruns@hereon.de) and Berit Zeller-Plumhoff (berit.zeller-plumhoff@hereon.de).


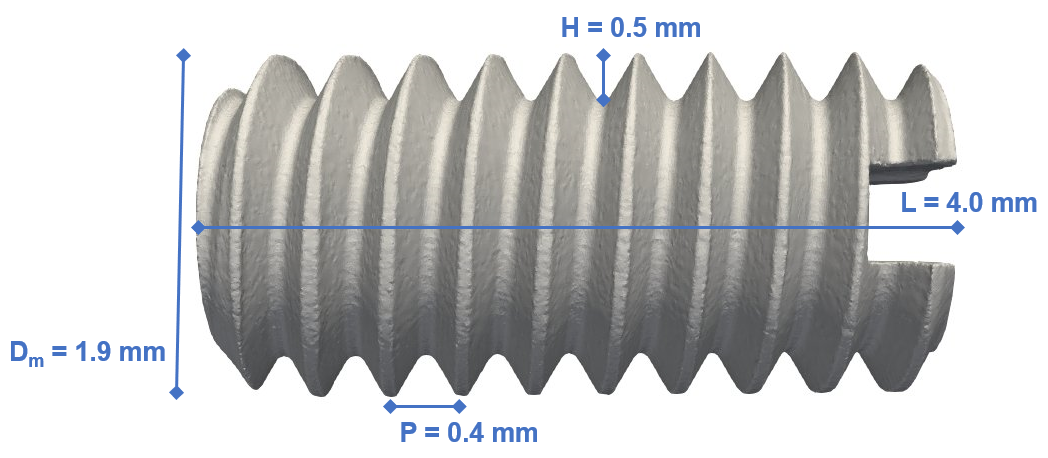


**Figure SI1:** Rendering of a titanium screw reconstructed and segmented from a µ-CT scan of a bone­–implant explant illustrating the design parameters of the screws employed in this study: major diameter (**D_m_**), screw length (**L**), thread pitch (**P**) and thread height (**H**).


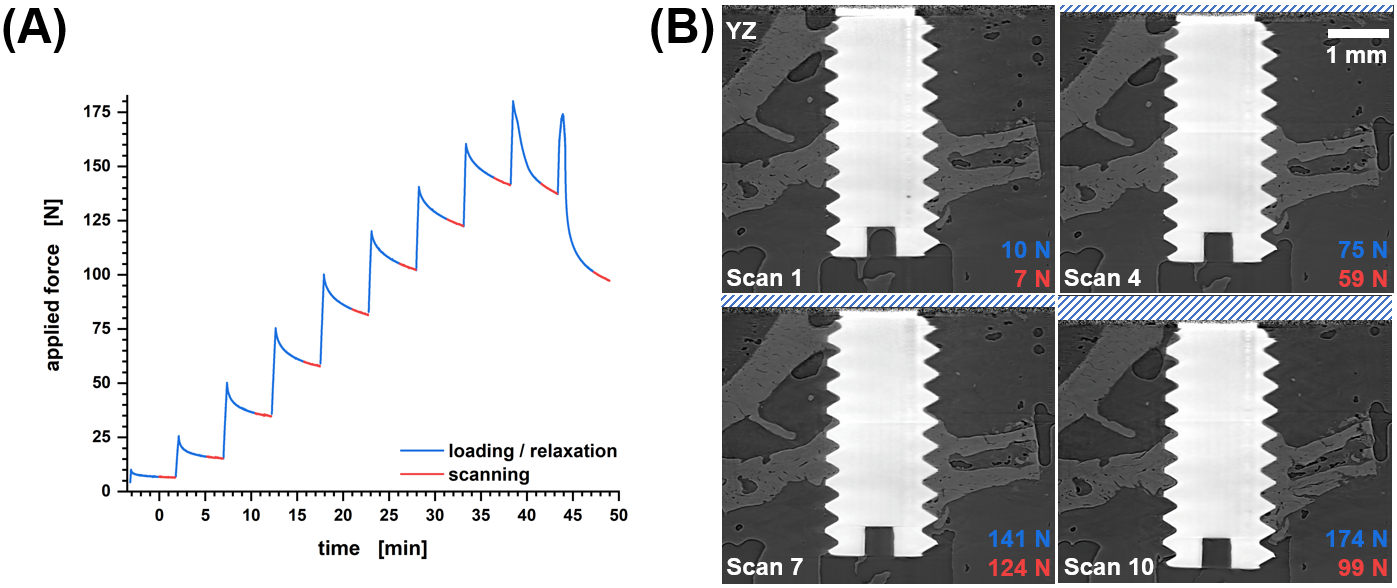


**Figure SI2:** (**A**) Profile of the applied force for an explant with a titanium screw acquired after twelve weeks of healing as an exemplary push-out experiment and (**B**) YZ-slices through the reconstructed volume after initial registration with respect to the sample holder and at different loading steps. The pin pushing the screw downwards is visible in the top part of the orthoslices with the dashed area outlining the covered distance. Blue values denote the maximal recorded force before relaxation, red values provide the mean force during scan time.


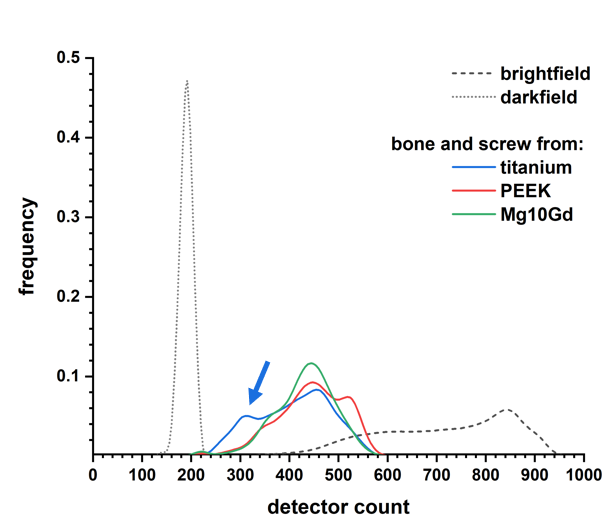


**Figure SI3:** Histogram of detector counts for brightfield, darkfield and sample projections with experimental parameters optimized for dosage. The blue arrow indicates the detector count of ~310 expected through a titanium screw which is only 1.6 times the count in the darkfield.


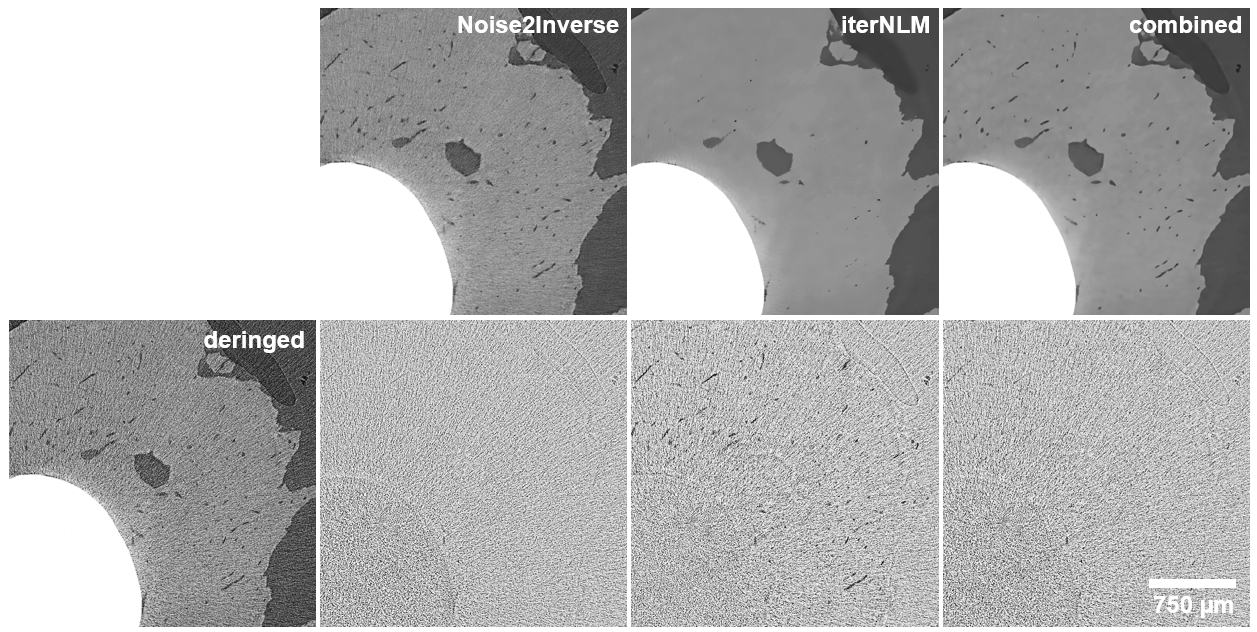


**Figure SI4:** Comparison of denoising approaches (top row) and difference to the reconstructed slice after ring removal (bottom row). Machine learning based filtering with a Noise2Inverse-filter cannot address correlated streak artefacts efficiently (2^nd^ column). Iterative Non-local means filtering (iterNLM) can be used to suppress these undesired textures but results in a loss of detail when applied to an image with high noise level (3^rd^ column). Combining the filters by first reducing the overall noise level with Noise2Inverse and shrinking textured artefacts with iterNLM subsequently preserves most details and reduces streak artefacts efficiently (right column).


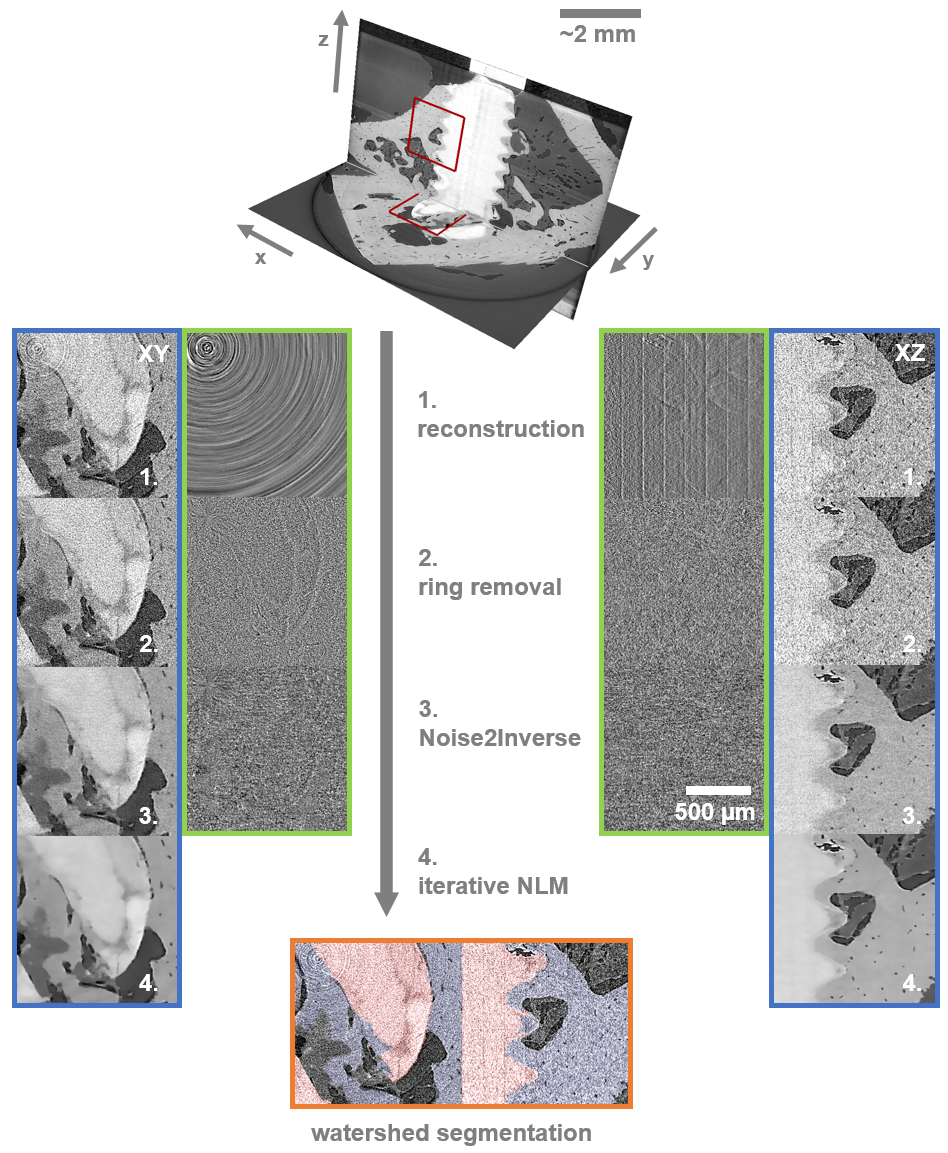


**Figure SI5:** Outline of the applied image processing routine. Indicated by the red frames are detail views of a scan recorded from an explant with a Mg-10Gd screw implant (acquired after a healing period of twelve weeks) in the reconstructed xz-plane and parallel to the axis of rotation (xy). Individual processing steps (blue frame) are shown alongside with difference images to the next processing step (green frame). Colour coded images in the orange frame overlay the segmentation into implant material (red), bone (blue) and void phase (transparent) over the initial reconstruction view. Note how the implant surface has become jagged with fully degraded regions being replaced by bone phase.

**Table SI1:** Comparison of bulk morphological parameters estimated in this study with values determined by Krüger et al..^[1]^ Parameters were determined according to Krüger et al., i.e., BV/TV was calculated in a volume of interest around the implant (r_VOI_) and BIC was evaluated including the surface of the screw implant protruding out of the cortical bone. Both measures were evaluated for their mean and standard deviation (σ). p-values are given for one-tailed unpaired two sample T-tests evaluating the difference between population means.

|  |  | | |  |  | **This study** | | | **Krüger *et al.* [1]** | | |  |
| --- | --- | --- | --- | --- | --- | --- | --- | --- | --- | --- | --- | --- |
| **Measure** | | **Material** | **Time** | | **r_VOI_ [µm]** | **Mean** | **σ** | **N** | **Mean** | **σ** | **N** | **p** |
| BV/TV | Titanium | | 4 weeks | | 100 | 0.495 | 0.032 | 6 | 0.523 | 0.079 | 5 | 0.247 |
|  |  | |  | | 200 | 0.452 | 0.021 | 6 | 0.488 | 0.104 | 5 | 0.246 |
|  |  | |  | | 300 | 0.441 | 0.031 | 6 | 0.473 | 0.109 | 5 | 0.282 |
|  |  | | 8 weeks | | 100 | 0.497 | 0.046 | 5 | 0.591 | 0.046 | 8 | **0.003*** |
|  |  | |  | | 200 | 0.435 | 0.043 | 5 | 0.513 | 0.046 | 8 | **0.006*** |
|  |  | |  | | 300 | 0.408 | 0.040 | 5 | 0.475 | 0.043 | 8 | **0.009*** |
|  |  | | 12 weeks | | 100 | 0.516 | 0.056 | 6 | 0.550 | 0.069 | 8 | 0.165 |
|  |  | |  | | 200 | 0.464 | 0.048 | 6 | 0.491 | 0.077 | 8 | 0.218 |
|  |  | |  | | 300 | 0.438 | 0.047 | 6 | 0.462 | 0.078 | 8 | 0.244 |
|  | PEEK | | 4 weeks | | 100 | 0.350 | 0.033 | 3 | 0.335 | 0.069 | 9 | 0.310 |
|  |  | |  | | 200 | 0.344 | 0.036 | 3 | 0.365 | 0.077 | 9 | 0.272 |
|  |  | |  | | 300 | 0.333 | 0.042 | 3 | 0.383 | 0.078 | 9 | 0.102 |
|  |  | | 8 weeks | | 100 | 0.421 | 0.061 | 3 | 0.455 | 0.066 | 10 | 0.230 |
|  |  | |  | | 200 | 0.410 | 0.051 | 3 | 0.428 | 0.058 | 10 | 0.318 |
|  |  | |  | | 300 | 0.403 | 0.040 | 3 | 0.413 | 0.057 | 10 | 0.372 |
|  |  | | 12 weeks | | 100 | 0.489 | 0.047 | 5 | 0.591 | 0.046 | 9 | **0.002*** |
|  |  | |  | | 200 | 0.445 | 0.035 | 5 | 0.494 | 0.057 | 9 | **0.036*** |
|  |  | |  | | 300 | 0.427 | 0.035 | 5 | 0.475 | 0.054 | 9 | **0.035*** |
|  | Mg-10Gd | | 4 weeks | | 100 | 0.282 | 0.027 | 2 | 0.259 | 0.101 | 8 | 0.296 |
|  |  | |  | | 200 | 0.278 | 0.007 | 2 | 0.251 | 0.085 | 8 | 0.204 |
|  |  | |  | | 300 | 0.286 | 0.002 | 2 | 0.263 | 0.082 | 8 | 0.231 |
|  |  | | 8 weeks | | 100 | 0.371 | 0.006 | 3 | 0.38 | 0.045 | 9 | 0.290 |
|  |  | |  | | 200 | 0.325 | 0.015 | 3 | 0.349 | 0.050 | 9 | 0.113 |
|  |  | |  | | 300 | 0.319 | 0.022 | 3 | 0.359 | 0.050 | 9 | **0.045*** |
|  |  | | 12 weeks | | 100 | 0.417 | 0.105 | 5 | 0.515 | 0.058 | 8 | 0.054 |
|  |  | |  | | 200 | 0.351 | 0.087 | 5 | 0.476 | 0.062 | 8 | **0.014*** |
|  |  | |  | | 300 | 0.333 | 0.078 | 5 | 0.467 | 0.063 | 8 | **0.007*** |
|  | Mg-5Gd | | 4 weeks | | 100 | 0.208 | 0.008 | 3 | 0.235 | 0.043 | 9 | 0.054 |
|  |  | |  | | 200 | 0.202 | 0.012 | 3 | 0.236 | 0.039 | 9 | **0.021*** |
|  |  | |  | | 300 | 0.205 | 0.019 | 3 | 0.248 | 0.04 | 9 | **0.019*** |
|  |  | | 8 weeks | | 100 | N/A | N/A | N/A | 0.405 | 0.042 | 10 | N/A |
|  |  | |  | | 200 | N/A | N/A | N/A | 0.365 | 0.043 | 10 | N/A |
|  |  | |  | | 300 | N/A | N/A | N/A | 0.359 | 0.045 | 10 | N/A |
|  |  | | 12 weeks | | 100 | 0.414 | 0.037 | 2 | 0.495 | 0.054 | 6 | 0.055 |
|  |  | | |  | 200 | 0.390 | 0.067 | 2 | 0.452 | 0.043 | 6 | 0.199 |
|  |  | | |  | 300 | 0.380 | 0.069 | 2 | 0.444 | 0.034 | 6 | 0.204 |
| BIC | Titanium | | | 4 weeks | N/A | 0.620 | 0.188 | 6 | 0.377 | 0.150 | 5 | **0.021*** |
|  |  | | | 8 weeks | N/A | 0.754 | 0.117 | 5 | 0.573 | 0.104 | 8 | **0.011*** |
|  |  | | | 12 weeks | N/A | 0.669 | 0.175 | 6 | 0.511 | 0.149 | 8 | 0.053 |
|  | PEEK | | | 4 weeks | N/A | 0.145 | 0.032 | 3 | 0.075 | 0.038 | 9 | **0.017*** |
|  |  | | | 8 weeks | N/A | 0.181 | 0.029 | 3 | 0.195 | 0.055 | 10 | 0.284 |
|  |  | | | 12 weeks | N/A | 0.246 | 0.135 | 5 | 0.272 | 0.056 | 9 | 0.348 |
|  | Mg-10Gd | | | 4 weeks | N/A | 0.468 | 0.009 | 2 | 0.360 | 0.130 | 8 | **0.026*** |
|  |  | | | 8 weeks | N/A | 0.586 | 0.169 | 3 | 0.48 | 0.078 | 9 | 0.196 |
|  |  | | | 12 weeks | N/A | 0.636 | 0.115 | 5 | 0.543 | 0.065 | 8 | 0.076 |
|  | Mg5-Gd | | | 4 weeks | N/A | 0.363 | 0.137 | 3 | 0.25 | 0.077 | 9 | 0.143 |
|  |  | | | 8 weeks | N/A | N/A | N/A | N/A | 0.492 | 0.063 | 10 | N/A |
|  |  | | | 12 weeks | N/A | 0.476 | 0.003 | 2 | 0.531 | 0.104 | 6 | **0.042*** |

* null hypothesis of equal population means rejected at 5% significance level

[1] D. Krüger, S. Galli, B. Zeller-Plumhoff, D. C. F. Wieland, N. Perruzzi, B. Wiese, P. Heuser, J. Moosmann, A. Wennerberg, R. Willumeit-Römer, *Bioact. Mater.* **2022**, *13*, 37–52, DOI: [10.1016/j.bioactmat.2021.10.041](https://doi.org/10.1016/j.bioactmat.2021.10.041).


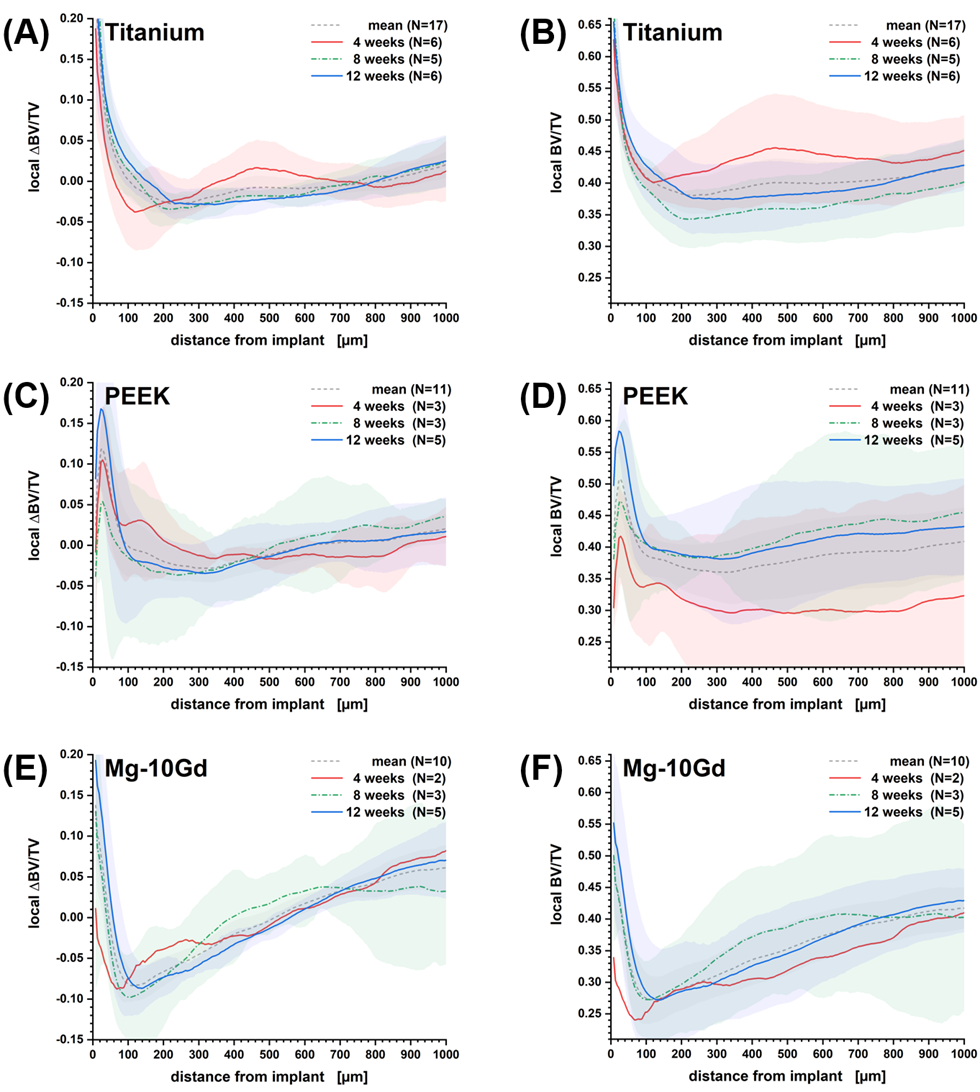


**Figure SI6:** Local change in mean bone volume fraction for different screw materials and timepoints after implantation with **(A,C,E)** respect to the bone volume fraction within 1 mm of the implant for three different timepoints after implantation visualizing the expected deviation for peri-implant BV/TV from the average BV/TV found in the ROI. Panels **B,D,F** visualize the local expected values in absolute terms but with higher uncertainty. Shaded areas provide the t-score based 95% confidence interval for all samples with N > 2.


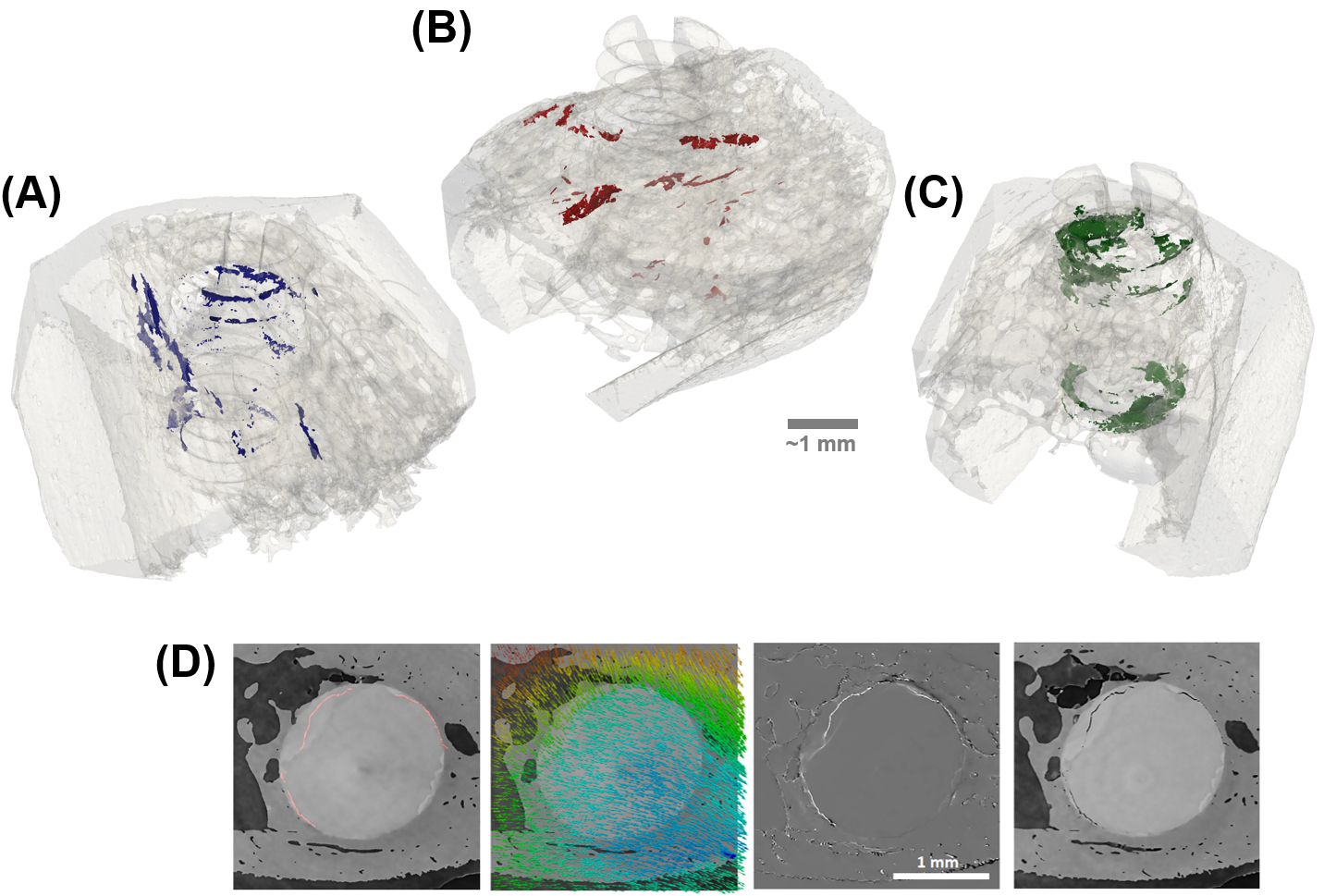


**Figure SI7:** Rendering of cracks identified in explants with a twelve-week healing period. Cracks were identified with a heuristic ruleset from dense volumetric strain fields calculated for the loading step before catastrophic failure. (**A**) Displayed are a bone–screw system with a titanium implant showing longitudinal cracks in the cortical bone and detachments at the bone–screw interface in blue. (**B**) An explant with a PEEK screw did not show detachments but additional cracks in the trabecular bone phase shown in red. (**C**) The Mg-5Gd screw system on the right collapsed in the corroded regions of the implant (green cracks) leaving the bone tissue mostly unaffected. (**D**) Slices through the reconstruction illustrate the crack recovery in 2D: the scan under load (right) is mapped onto a scan of the relaxed sample (left) to calculate a displacement vector field (centre left) yielding positive volumetric strains (centre right) that were used to locate the position of cracks in the initial morphology (superimposed in red).


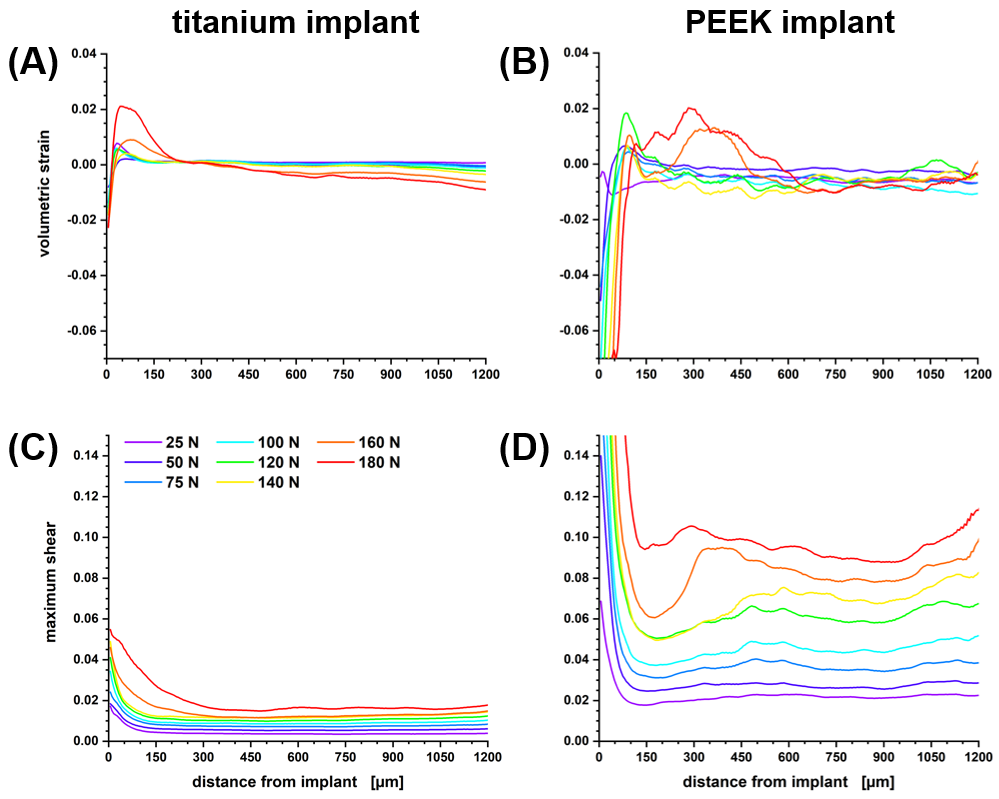


**Figure SI8:** Complementary information to Figure 5 in the main text showing (**A**,**B**) a comparison of the volumetric strain and (**C**,**D**) the maximum shear strain as a function of the distance to the implant for the peri-implant bone in the evaluated titanium implant system (left panels) and PEEK implant system (right panels).

***
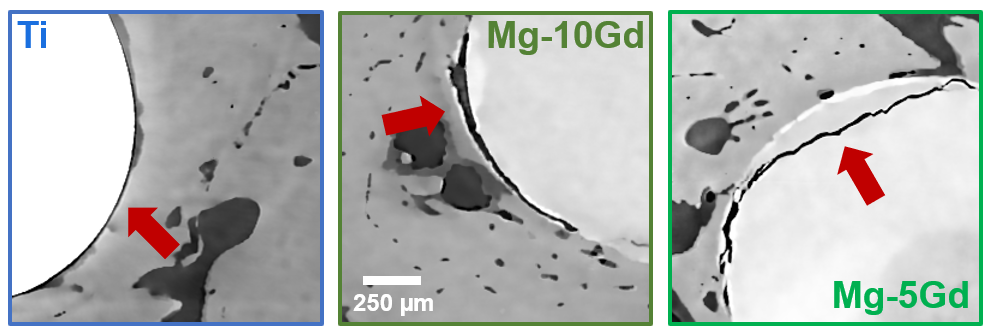
***

**Figure SI9:** Examples of bone tissue detaching from a metal implant. With titanium implants, detachment occurs as adhesive failure (left) whereas metal residuals can be identified as bright spots in the detached tissue with magnesium-gadolinium implants (center). The alloy-corrosion interface may also provide an additional mechanical weak point for implant failure (right).


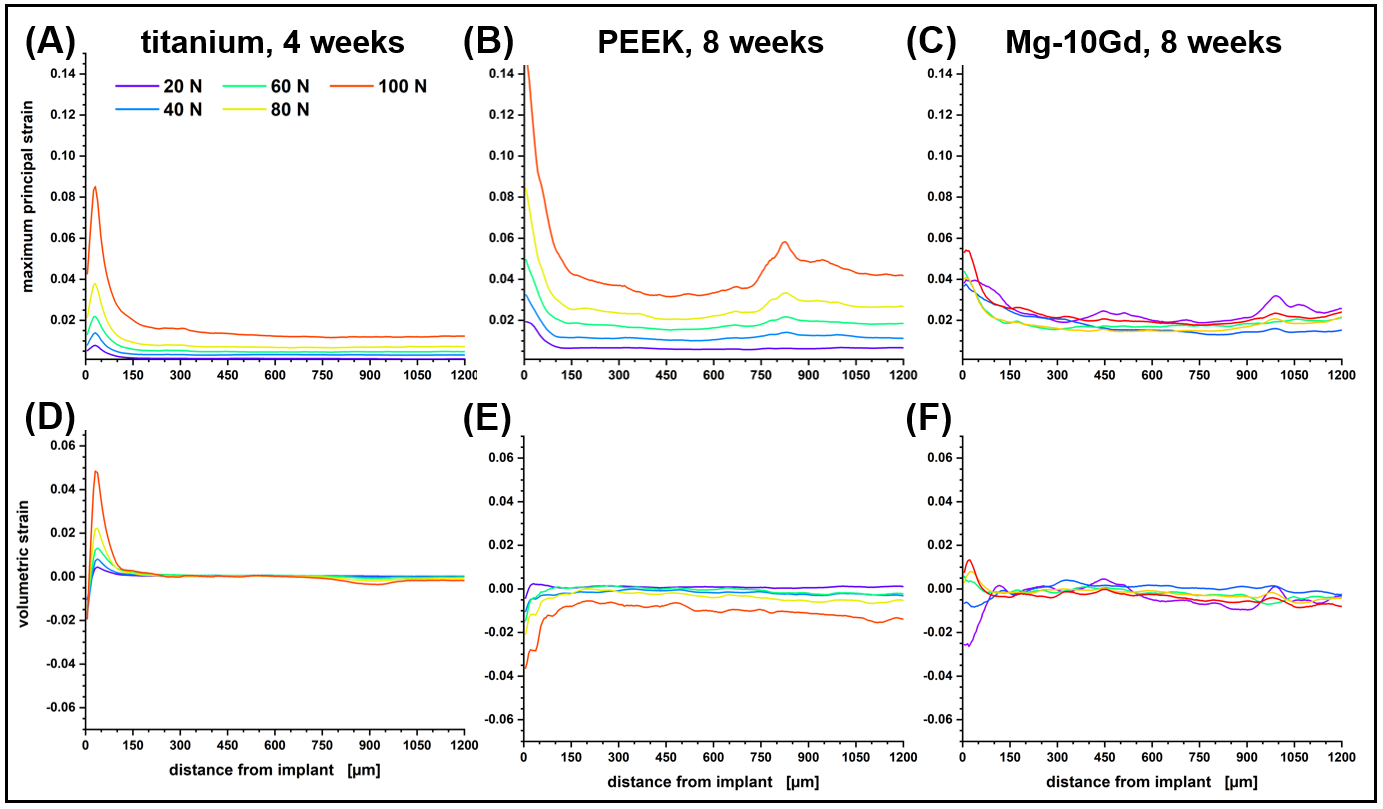


**Figure SI10:** Comparison of (**A**–**C**) maximum principal strain and **(D**–**F**) volumetric strain as a function of the distance to the implant for titanium implants (left panels), PEEK implants (central panels) and Mg-10Gd implants (right panels). All three implants failed at an applied push-out load of approximately 120 N.

**Table SI2:** Expected values for nominal stiffness depending on material and healing period. Samples lacking a quality reconstruction for morphological parameter estimation were excluded from the correlative analysis in the main text. This applies to two PEEK samples with four-week healing period, one Mg-10Gd sample with twelve-week healing period and one Mg-5Gd sample with twelve-week healing period.

| **Measure** | | **Material** | **Time** | | **Mean** | **σ** | **N** |
| --- | --- | --- | --- | --- | --- | --- | --- |
| k_nom_ | Titanium | | | 4 weeks | 468 | 149 | 6 |
| [N/mm] |  | | | 8 weeks | 519 | 181 | 5 |
|  |  | | | 12 weeks | 610 | 206 | 6 |
|  |  | | | any | 533 | 180 | 17 |
|  | PEEK | | | 4 weeks | 295 | 119 | 3 |
|  |  | | | 8 weeks | 265 | 55.2 | 3 |
|  |  | | | 12 weeks | 360 | 97.1 | 5 |
|  |  | | | any | 316 | 95.3 | 11 |
|  | Mg-10Gd | | | 4 weeks | 177 | 49 | 2 |
|  |  | | | 8 weeks | 401 | 183 | 3 |
|  |  | | | 12 weeks | 490 | 150 | 5 |
|  |  | | | any | 400 | 183 | 10 |
|  | Mg5-Gd | | | 4 weeks | 138 | N/A | 1 |
|  |  | | | 8 weeks | N/A | N/A | 0 |
|  |  | | | 12 weeks | 291 | N/A | 1 |
|  |  | | | any | 214 | 108 | 2 |
|  | any | | | 4 weeks | 349 | 176 | 12 |
|  |  | | | 8 weeks | 418 | 181 | 11 |
|  |  | | | 12 weeks | 483 | 186 | 17 |
|  |  | | | any | 425 | 186 | 40 |


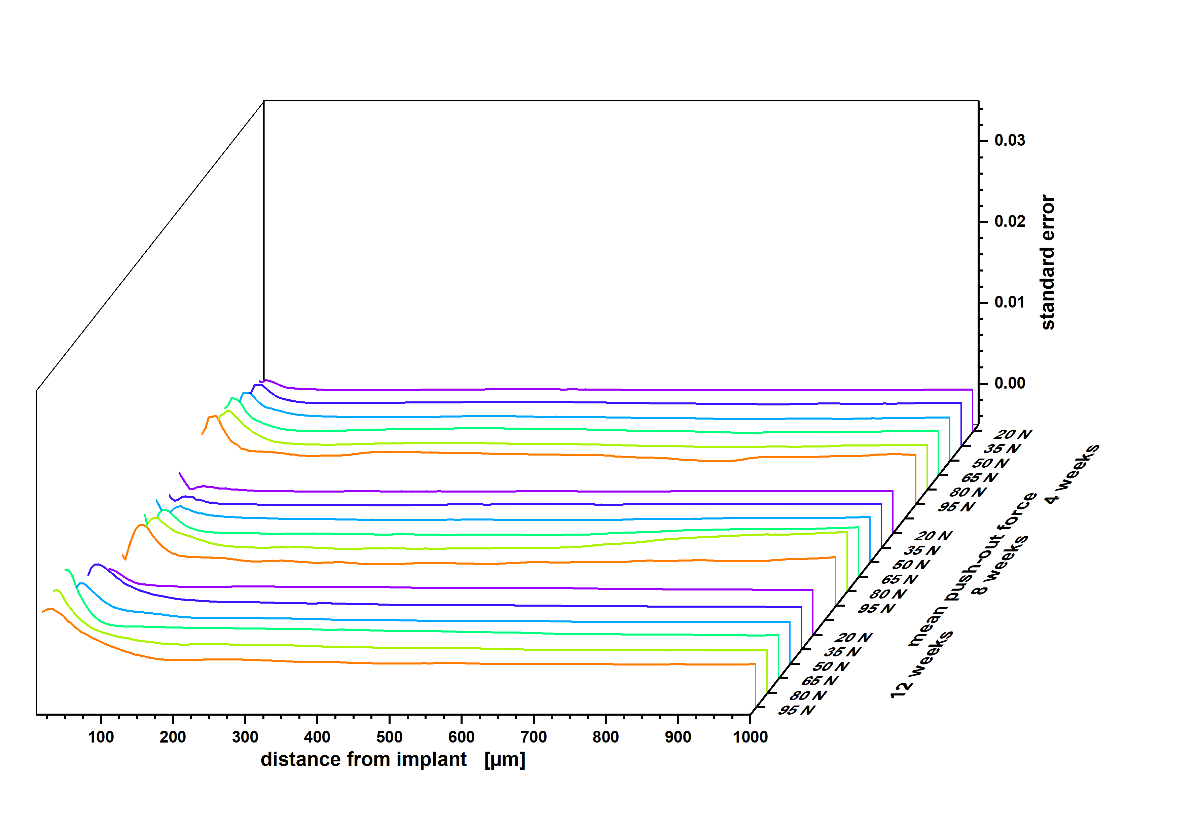


**Figure SI11:** Standard error of mean volumetric strain for peri-implant bone of titanium implant systems with respect to healing period and applied force during scan time (color). Associated mean values are shown in **Figure 4**.


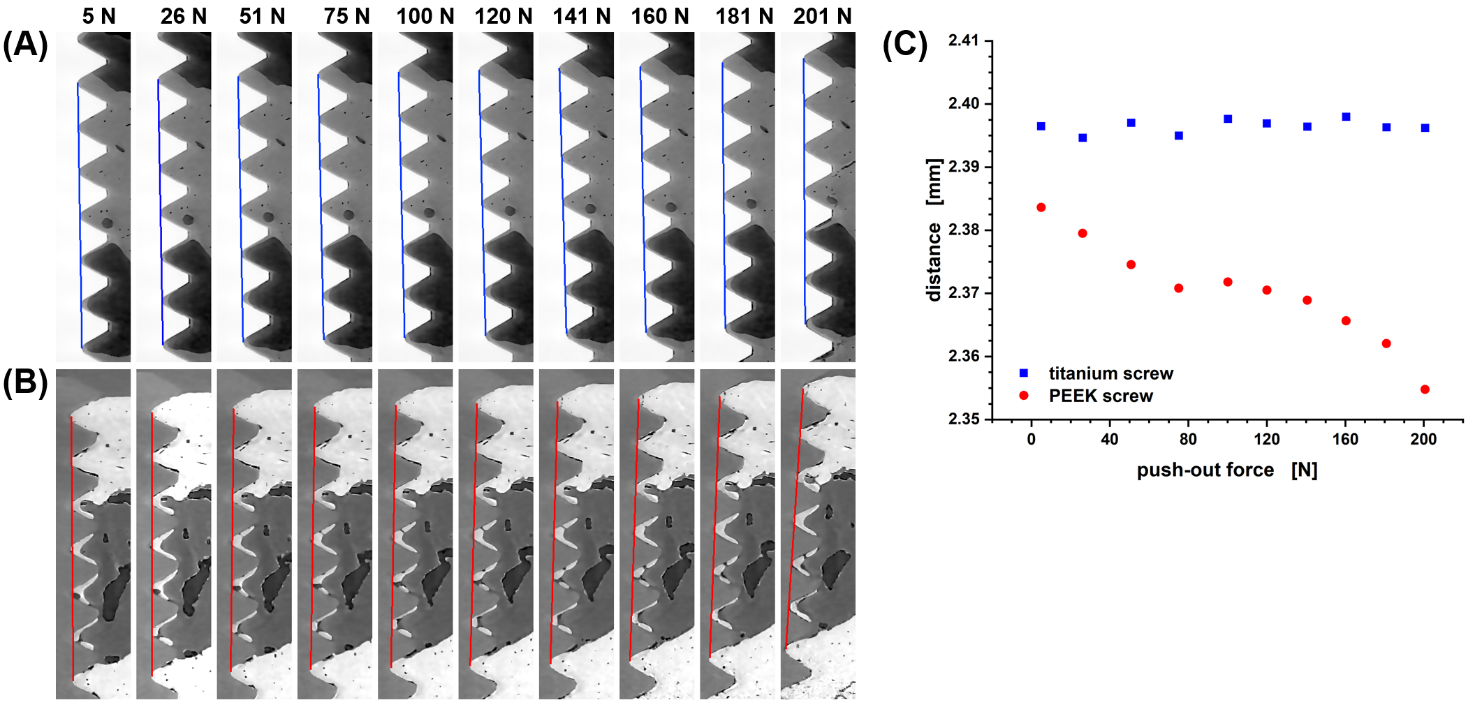


**Figure SI12:** Evaluation of screw compression for samples shown in **Figure 5**. The distance across six threads was measured at increasing load for **(A)** a reference titanium screw (blue lines) **(B)** and a PEEK screw (red lines). **(C)** The plot reveals that the PEEK screw is compressed by 29 µm or 1.2% over the course of the experiment whereas the distance measured for the titanium screw remained constant.
